# Quantitative Dynamics of Telomere Bouquet Formation

**DOI:** 10.1371/journal.pcbi.1002812

**Published:** 2012-12-06

**Authors:** David M. Richards, Emma Greer, Azahara C. Martin, Graham Moore, Peter J. Shaw, Martin Howard

**Affiliations:** 1 Computational and Systems Biology, John Innes Centre, Norwich Research Park, Norwich, Norfolk, United Kingdom; 2 Cell & Developmental Biology, John Innes Centre, Norwich Research Park, Norwich, Norfolk, United Kingdom; 3 Crop Genetics, John Innes Centre, Norwich Research Park, Norwich, Norfolk, United Kingdom; University of Washington, United States of America

## Abstract

The mechanism by which homologous chromosomes pair during meiosis, as a prelude to recombination, has long been mysterious. At meiosis, the telomeres in many organisms attach to the nuclear envelope and move together to form the telomere bouquet, perhaps to facilitate the homologous search. It is believed that diffusion alone is not sufficient to account for the formation of the bouquet, and that some directed movement is also required. Here we consider the formation of the telomere bouquet in a wheat-rye hybrid both experimentally and using mathematical modelling. The large size of the wheat nucleus and wheat's commercial importance make chromosomal pairing in wheat a particularly interesting and important process, which may well shed light on pairing in other organisms. We show that, prior to bouquet formation, sister chromatid telomeres are always attached to a hemisphere of the nuclear membrane and tend to associate in pairs. We study a mutant lacking the *Ph1* locus, a locus ensuring correct homologous chromosome pairing, and discover that bouquet formation is delayed in the wild type compared to the mutant. Further, we develop a mathematical model of bouquet formation involving diffusion and directed movement, where we show that directed movement alone is sufficient to explain bouquet formation dynamics.

## Author Summary

The appearance of sexual reproduction over a billion years ago led to a revolution in how organisms pass on genetic material to their offspring. In sexually reproducing organisms parental diploid cells, containing two nearly identical copies of each chromosome (homologues), produce gametes containing only one copy of each chromosome. This in turn requires the pairing of the related homologous chromosomes to ensure their subsequent segregation into the gametes. How this pairing is achieved is poorly understood since chromosomes must search the entire nucleus for their homologous partner. Many organisms move the ends of each chromosome (the telomeres) along the periphery of the nucleus into a small patch forming the telomere bouquet. We show here that direct movement of telomeres towards the bouquet site, potentially driven by molecular motors, can explain bouquet formation dynamics. We focus in particular on a wheat-rye hybrid since understanding homologous pairing in wheat could have profound implications for breeding resistant crops by aiding the production of hybrids. We also show that wheat seems to have evolved a mechanism to delay the onset of telomere bouquet formation, perhaps in order to ensure chromosomes find their correct homologous partners.

## Introduction

Meiosis, an integral component of the mechanism of sexual reproduction, is a crucial process in eukaryotes, resulting in a halving of the number of chromosomes. Such a process allows genetic material to be shared during fertilisation, whilst maintaining the same amount of DNA per cell. Diploid cells that undergo meiosis must first pair homologous chromosomes so that gametes can be formed containing only one copy of each pair. The mechanism by which pairing occurs has long been an outstanding problem [1], [2] since thermally-driven diffusion of chromosomes is probably much too slow to ensure pairing in the observed time scale of hours [2].

Many organisms attach telomeres to the nuclear membrane before pairing, although how this is achieved is largely mysterious. Further, many of these organisms then move the telomeres together, until they form one cluster on the membrane producing the telomere bouquet [3], [4], [5], [6], [7], [8]. This is in contrast to the Rabl configuration, seen in some organisms during interphase, where centromeres and telomeres occupy opposite sides of the nucleus. In addition there is often complex motion of the entire chromatin as, for example, has been observed in maize [9]. It has been suggested that the telomere bouquet facilitates homologous pairing, perhaps by reducing the search space to a much smaller region. The method by which the bouquet forms is not well understood. However, it is known that numerous organisms contain pairs of SUN-KASH proteins, which link chromosomes to cytoskeletal motors, and potentially these motors could pull the telomeres around the nuclear membrane [10].

Although chromosome motion has been much studied during mitosis, there are far fewer mathematical and computational models of chromosome organisation during meiosis. A purely mathematical model of homology searching was performed in [11] where, along with comparing a 2D search along the nuclear membrane with a 3D full nucleus search, the effect of the number of homology recognition sites per chromosome was analysed. Recently, the effect of the telomere bouquet on homologous pairing has been modelled, in an attempt to understand the reason for bouquet formation [12]. Another recent paper studied the spatial organisation of meiotic chromosomes after pairing is complete, when homologous chromosomes are arranged in synaptonemal complexes [13]. A combination of experiments and modelling in rye were used in [14] where it was argued that directed movement of telomeres is required to form the bouquet in the observed time. However, diffusion was also needed to explain all their experimental data. Here we significantly extend this earlier work and our general understanding of meiosis both experimentally and theoretically, demonstrating the novel result that directed movement alone (without diffusion) can in fact explain bouquet formation dynamics. We also examine the degree of variation in this directed movement, presumably due to disorder in the appropriate underlying cytoskeletal elements. In addition we determine for the first time the initial arrangement of telomeres on the nuclear envelope, before telomere bouquet formation begins.

The problem of chromosome pairing in wheat is particularly acute due to both the large nuclear radius (∼8 µm) and the relatively large chromosomes (average of ∼800 Mb each). These should be compared to typical values in, say, the yeast *S. cerevisiae*, where the nuclear radius is around 1 µm and the average chromosome size is less than 1 Mb. Although it might be thought that the much larger nuclear volume in wheat would drastically increase the pairing time, wheat is able to complete pairing in times similar to those for other organisms. Wheat may achieve this feat by utilising a greater level of meiotic chromosome organisation. In addition to clustering all telomeres in a bouquet as in many organisms, wheat carefully controls the centromere positions, both by maintaining the Rabl configuration during interphase and by forming seven centromere clusters shortly before the telomere bouquet is formed [15]. However, since these centromere clusters form when the telomere bouquet is almost complete, it is unlikely that they significantly influence the dynamics of bouquet formation. Similar features are found in bouquet formation in maize, where centromeres and telomeres are both organised during meiosis [8].

Pairing in bread wheat is further complicated by its hexaploid nature where, due to hybridisation of diploid ancestors, the genetic material consists of three closely related genomes. With each nucleus containing six related copies of each chromosome, it is important to ensure that pairing only occurs between homologous pairs. This pairing specificity has been shown to involve the *Ph1* (Pairing homeologous 1) locus, a region located on chromosome 5B. Deletion of this region leads to homeologous pairs (i.e. related but non-homologous pairs), chromosome rearrangements, and eventually infertility. The *Ph1* locus has been defined to a cluster of defective Cdk-like genes that have been shown to suppress Cdk-2 type activity and hence histone H1 phosphorylation [16].

To examine homologous chromosome pairing and bouquet formation we studied two wheat-rye hybrids: a wild type containing the *Ph1* locus and a mutant where *Ph1* has been deleted. Sexual hybridization between wheat and a wild relative generally produces an interspecific hybrid containing a haploid set of related but non-homologous chromosomes (homeologues), in which chromosome pairing is largely prevented as a result of the presence of *Ph1* [17]. Although non-homologous pairing is prevented, the telomere bouquet still forms even in a wheat-rye hybrid containing *Ph1* [16]. Since pairing is normally prevented, wheat-rye hybrids are ideal for studying the *Ph1* locus: the presence of paired chromosomes can then be used as an easily identified phenotypic signal of unusual *Ph1* behaviour. Further, understanding the basis for pairing suppression may lead to the important practical application of being able to switch the pairing on and off, thereby enhancing breeding strategies [18].

In this study, we analyse both the initial distribution of telomeres (after telomeres have moved to the nuclear envelope but prior to bouquet formation) and their dynamics as they form the bouquet. We show that, before bouquet formation, sister chromatid telomeres are always attached to a randomly-orientated hemisphere of the nuclear envelope and tend to associate in pairs. We combine fluorescence microscopy with mathematical modelling to shed light on how telomeres move along the nuclear membrane, how the bouquet forms and the relative roles of diffusion and directed movement. Further, we study the differences between plants with and without the *Ph1* locus, showing that bouquet formation is delayed in the presence of *Ph1*.

## Results

### Data collection

Wheat–rye hybrids have a haploid set of 21 wheat chromosomes and seven rye chromosomes. Replication of the rye heterochromatin knobs can be easily visualized in these lines. Previous data showed that in wheat-rye hybrids, either with or without *Ph1*, DNA replication is initiated in the meiocytes as the tapetal cells are finishing their replication, and all meiocyte replication is completed within a 4 hr period before the telomeres form the bouquet [16]. Therefore, DNA replication in the meiocytes is a good guide for identifying the onset of telomere dynamics and bouquet formation.

**Figure 1:**
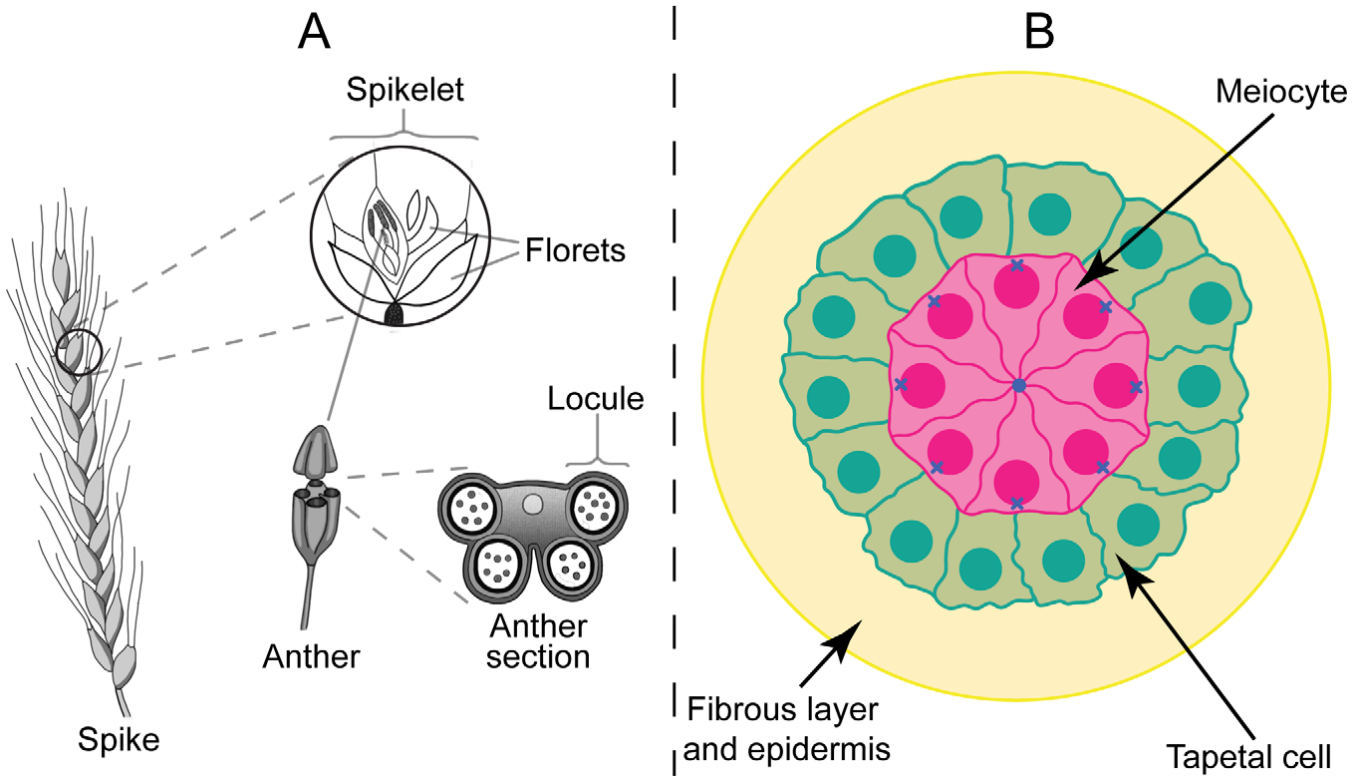
Structure of a wheat spike and anther, showing the position of the meiocytes. A. Sketch of a wheat spike, showing spikelets, florets and anthers. Each anther contains four locules. B. Sketch of the cross section of one of the locules within a wheat anther, with meiocytes in pink, tapetal cells in green and the remaining cells in yellow (not shown in detail). Solid circles represent nuclei and blue crosses show the outside pole of each meiocyte nucleus, where the telomere bouquet normally forms.

**Figure 2:**
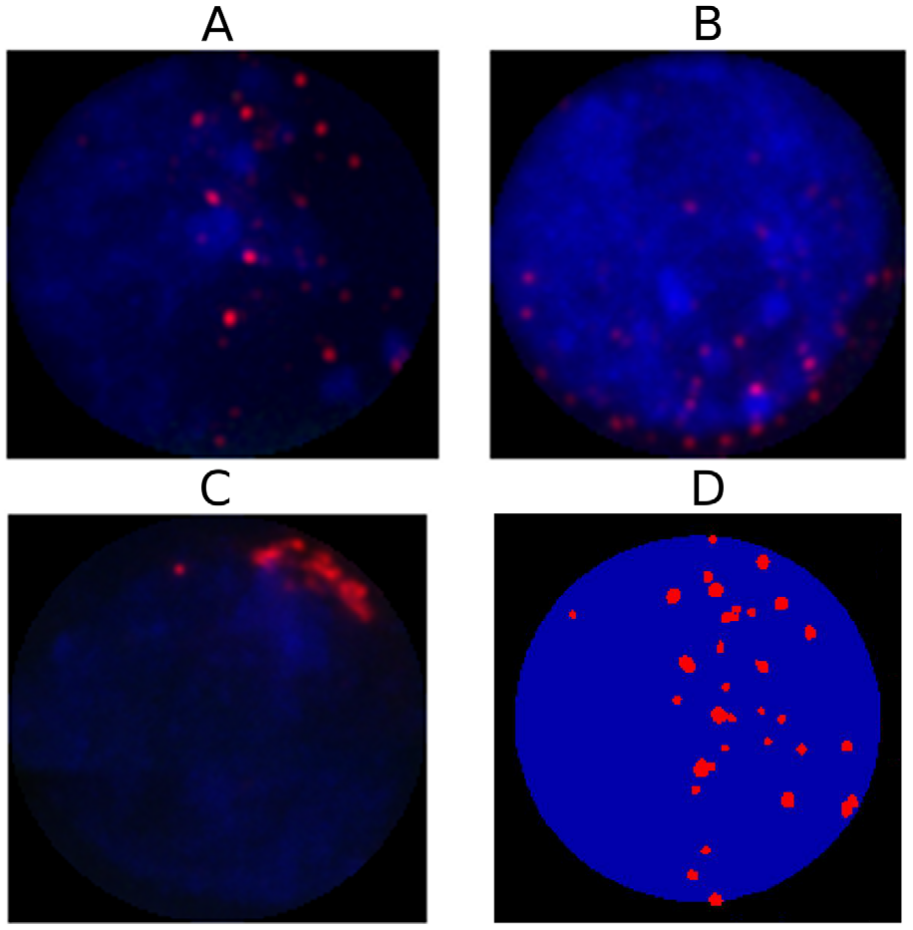
Experimental *Ph1*− meiocyte images and the associated identification of telomere positions. *Ph1*+ and tapetal nuclei look almost identical although tapetal cells do not form a telomere bouquet. A–C. Three separate *Ph1*− meiocyte nuclei showing different stages of bouquet formation. Each image has been sum-projected over z-stacks, with the DAPI stain shown in blue and the telomeres labelled in red. D. Data from A after the nucleus (in blue) and telomere clusters (in red) have been identified by our image processing.

In this work, DNA replication in wheat–rye anthers was analyzed by diffusing in 5-ethynyl-2′-deoxyuridine (EdU), a nucleoside analog of thymidine that is incorporated into DNA during active DNA synthesis. In EdU, the terminal methyl group is replaced with an alkyne group, which allows detection using a fluorescent azide compound that covalently binds to the alkyne group in a “click chemistry” reaction [19]. This technology is quick, very specific, and does not require DNA denaturation. It therefore provides good structural preservation and is compatible with dual labelling with telomere probes. After EdU treatment for 4 hr, anthers of wheat-rye hybrids (see Figure 1A), both with and without the *Ph1* locus in the wheat genome, were labelled with a telomere probe by fluorescence in situ hybridization (FISH) and chromosomes were counterstained with 4′,6-diamidino-2-phenylindole (DAPI), as described in detail in *Materials and Methods*. Thus, anthers undergoing or just after DNA replication in the meiocytes were identified via EdU incorporation and labelling, while telomeres in the same meiocytes were labelled by FISH. Anther sections were then imaged using fluorescence microscopy. Our method offers a series of snapshots of *in vivo* states rather than potentially perturbing the normal progression by *in vitro* anther culture. These images were taken at essentially random times during (or just after) meiotic DNA replication. Each section gave rise to three stacks of 2D images: one stained with DAPI, one labelled with telomere probes and one labelled with EdU. The images include both meiocytes (which undergo meiosis to form gametes) and tapetal cells (which aid nutrient transportation within the anther and do not undergo meiosis); see Figure 1B. Figures 2A–C show example snapshots of the different stages of bouquet formation, ranging from dispersed telomere clusters to a tight telomere bouquet.

From the raw dataset of images we first extracted the positions of the telomere signals. In each case this involved using the DAPI image to determine the extent of the nucleus and the FISH telomere image to locate the telomeres. For each telomere signal we extracted its 3D position, its size and its intensity. Further, we recorded the point within each nucleus that was furthest from the centre of the anther, i.e. the point on the “outside” of the anther (Figure 1B). For details see *Materials and Methods* and the Supporting Information. Figure 2D shows a representative example of the cluster positions (extracted from the data in Figure 2A).

### Data analysis

Wheat-rye hybrid cells contain 21+7 chromosomes and so, after undergoing DNA replication, a meiocyte nucleus contains 112 telomeres, which, as telomeres at the same end of sister chromatids are close (or attached), would appear as 56 telomere signals. However, it is never possible (at least with our resolution) to even see 56 separate telomere signals. This is due to sister chromatid telomeres being close together (and perhaps even attached), forming sister chromatid telomere clusters. From now on we will often refer to sister chromatid telomeres, namely the pairing of the two telomeres at the same end of sister chromatids. When we refer to pairing/clustering of sister chromatid telomeres, we mean the pairing/clustering of these pairs of telomeres. So, for example, a pair of sister chromatid telomeres would involve a cluster of four telomeres in total.

**Figure 3:**
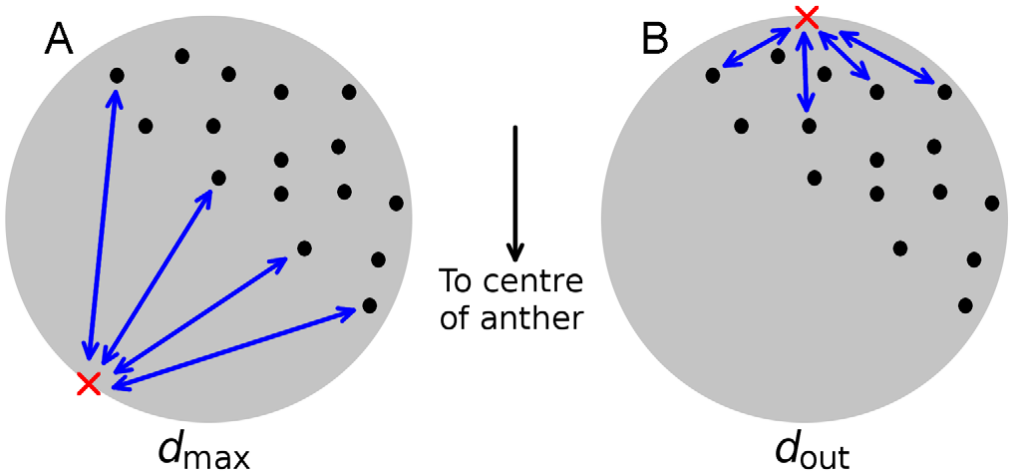
Sketch of the definitions of *d*_max_ and *d*_out_. Both quantities are the average distance between the telomere clusters and some other point weighted by the telomere cluster intensities, i.e. the weighted average of the lengths of the blue arrows. Although only some blue arrows are shown, the averages are taken over all telomere clusters. A. For *d*_max_ the relevant distance is that between the telomere clusters and the point on the nuclear envelope (red cross) that maximises the average distance. B. For *d*_out_ the relevant distance is that between the telomere clusters and the “outside” pole (red cross), i.e. the point on the nuclear envelope that is furthest from the centre of the anther.

From the positions and intensities of the telomere signals, we constructed three measures of the telomere distribution. First, we simply counted the number of telomere clusters in a given nucleus, which we call *N*. As telomere bouquet formation proceeds, *N* will gradually decrease as more and more telomeres join the bouquet. Second, we determined which point on the surface of the nucleus lies furthest from the telomere clusters (by maximising the sum of the 3D distances to the telomere clusters weighted by their intensities) and then recorded the average telomere distance from this point (again weighted by intensities). Figure 3A shows a sketch of the definition of *d*_max_. This distance, *d*_max_, always takes values above 1.2*R* (see Supporting Information) and, as bouquet formation proceeds, telomeres move closer to each other and *d*_max_ increases, reaching a maximum after bouquet formation has completed. Finally, we calculated *d*_out_, which is the average 3D distance from the telomere clusters to the “outside” pole of the nucleus weighted by intensity, where the “outside” pole of the nucleus is the point on the nuclear membrane furthest from the anther centre (Figure 1B). Figure 3B shows a sketch of how *d*_out_ is defined. Unlike *d*_max_ which increases with time, *d*_out_ decreases as bouquet formation proceeds, equalling zero only if the bouquet forms exactly on the outside of the nucleus. See Supporting Information for detailed definitions of *d*_max_ and *d*_out_.

Since our data are noisy, useful information can only be extracted by analysing many nuclei. We therefore considered the histogram of the number of telomere clusters, *N*, the histogram of the maximum average telomere cluster distance, *d*_max_, and the histogram of the average telomere cluster distance from the outside pole, *d*_out_. Although we do not have time-lapse images and so cannot track individual telomeres as they move towards the bouquet site, we can still extract information on bouquet formation dynamics by studying these histograms. For example, the cluster number histogram shows the proportion of time that nuclei spend with a given number of clusters, which is directly related to the telomere dynamics.

From the 3D positions of the telomere clusters, we checked that telomeres are attached to the nuclear envelope. To do so we calculated the distance of clusters from the centre of the nucleus as a fraction of the nuclear radius, and found that, in both meiocytes and tapetal cells (both with and without the *Ph1* locus), this normalised distance has average 0.9 and standard deviation 0.1. This supports the idea that telomeres are associated with the nuclear envelope, not just in meiocytes, but in tapetal cells and potentially, therefore, in many diverse cell types.

We also examined the position of the final bouquet in *Ph1*− meiocytes relative to the anther centre. To do this we studied images where the bouquet has completely or very nearly formed. In each case we measured the angle, from the centre of the nucleus, between the bouquet and the “outside” pole and plotted the histogram of these angles (Figure S1). If the bouquet formed at random positions on the nuclear membrane this would give a flat histogram. However, since Figure S1 is heavily weighted to small angles, this shows that, in the majority of cases, the bouquet tends to form close to the outside pole of the nucleus, the point on the nuclear membrane furthest from the centre of the anther (Figure 1B).

### Prior to bouquet formation, sister chromatid telomeres, on average, form pairs

During meiosis, meiocytes start with their telomeres attached to the nuclear membrane, and then gradually form the telomere bouquet by moving all telomeres to a small region on the membrane. However, before we study the dynamics of forming the bouquet, it is important to study the initial distribution of telomeres (after telomeres have attached to the nuclear envelope but before bouquet formation has started). This distribution can then be used to inform the initial condition for our mathematical model. Since it is not possible to determine from a still image whether a meiocyte has started bouquet formation, we instead use tapetal cells (both from *Ph1*+ and *Ph1*− plants) to study the initial telomere distribution in meiocytes. We also checked, as explained below, that the initial telomere distribution in meiocytes is indeed similar to the telomere distribution in tapetal cells.

As discussed previously, we never observed as many as 56 telomere clusters. In fact combining all our tapetal data we find, on average, only 27±1 telomere clusters (n = 133), where the error is the standard error of the mean number of telomere clusters. This suggests that there is always some association between sister chromatid telomeres. Furthermore, strikingly, 27 is close to half of 56, which suggests that the sister chromatid telomeres may be associating in pairs. We also confirmed this result separately for *Ph1*+ and *Ph1*− tapetal cells. There are three possibilities for how telomeres might pair together: sister chromatid telomeres on opposite ends of the same chromosome may pair, homeologous chromosomes may pair, or any two sister chromatid telomeres may pair non-specifically. It would be interesting to label individual chromosome telomeres to determine which of these associations occurs.

**Figure 4:**
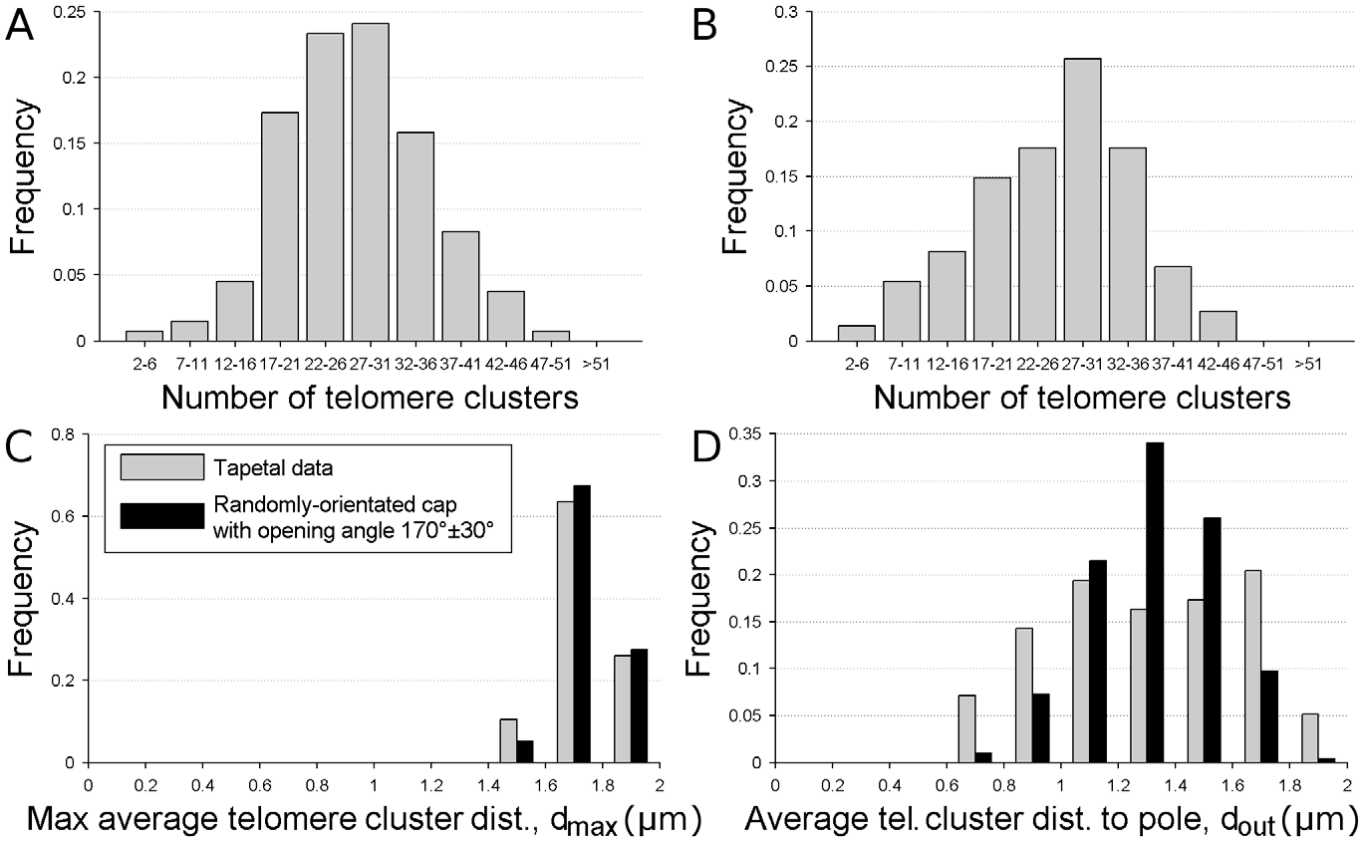
Histograms of the number of telomere clusters and histograms quantifying the telomere cluster spatial distribution. A. Distribution of the number of telomere clusters in tapetal cells (combining data both with and without the *Ph1* locus). n = 133, mean = 27.2±0.7, standard deviation = 8.1. B. Distribution of the number of telomere clusters in *Ph1*+ meiocytes. n = 74, mean = 25.9±1.0, standard deviation = 8.6. C. Distribution of maximum average telomere cluster distance, *d*_max_ (as a fraction of the nuclear radius), in tapetal cells (combining data with and without the *Ph1* locus, n = 98), compared to the theoretical situation where telomere clusters lie randomly positioned in a randomly-orientated cap subtending an opening angle of 170°±30°. D. As for C, but showing the distribution of average telomere cluster distance to the outside pole, *d*_out_ (as a fraction of the nuclear radius).

It is also revealing to study the distribution of the number of clusters. This distribution is approximately normal with a standard deviation of about 8 (see Figure 4A). This implies that, although sister chromatid telomeres on average form pairs, this is not always the case: sometimes fewer than 28 pairs form, and in other cases telomeres form clusters containing more than two sister chromatid telomeres. Potentially this could be because the association between nearby telomeres is transient, with only weak forces holding clusters together, so that telomeres can easily dissociate from existing clusters. It is also possible that the association is not between telomeres but between the chromosomes themselves, perhaps between subtelomeric regions. The exact region of the chromosomes that are associated would impact the distance that telomeres could move from each other, which could also explain the potential movement of telomeres in and out of clusters. Further, the fact that there are clusters containing more than two sister chromatid telomeres disfavours a model where the clusters are solely due to associations between opposite ends of single chromosomes. However, it would still be interesting to know whether pairings between telomeres at opposite ends of a chromatid pair are preferential. Again, labelling individual telomeres would help to answer this question.

### Telomere clusters are initially distributed in a randomly-orientated cap

In addition to the initial number of telomere clusters, we can also study the initial telomere cluster distribution in space. As before, the initial distribution refers to the distribution once the telomeres are attached to the nuclear envelope, but before bouquet formation has started. When chromosomes are in the Rabl configuration, with centromeres biased towards one side of the nucleus, it is often observed that the telomeres, being the regions on the chromosomes furthest from the centromeres, inhabit regions of the nucleus opposite to the centromeres. In wheat, the Rabl configuration persists through all stages of the cell cycle, and so we expect a bias in the initial distribution of telomeres. To study this we plot the histogram of the maximum average telomere cluster distance, *d*_max_, for tapetal cells (combining both *Ph1*+ and *Ph1*− cells; see Figure 4C). If telomere clusters were randomly distributed on the nuclear membrane, a computer simulation (see Supporting Information) shows that we would expect a histogram centred on 1.51*R*. Conversely if the telomere clusters were confined to a hemisphere the histogram would peak at 1.72*R*. Since the maximum in Figure 4C occurs at 1.74*R*, this suggests the telomere clusters are, on average, initially distributed within a region slightly more restricted than a hemisphere (which has an opening angle of 180°). This agrees with the examination of individual images, where it is often obvious that telomere clusters are preferentially grouped within some hemisphere (for example, see Figure 2D). In fact, simulations (see Supporting Information) show that this data fits well with telomere clusters confined to a cap subtending an opening angle that follows a normal distribution with mean <Θ_0_> = 170° and standard deviation δΘ_0_ = 30°. This result is not changed if *Ph1*+ or *Ph1*− tapetal cells are analysed separately.

To determine whether this initial cap has any preferred orientation with respect to the centre of the anther (as suggested in previous models [14]), we calculated the distribution of average distances of telomere clusters from the “outside” pole for tapetal cells, i.e. the distribution of *d*_out_ (Figure 4D). If, for example, telomere clusters sit in the “outside” cap, then the average value of *d*_out_ will be lower than for other distributions. In fact, computer simulations (see Supporting Information) show the average value of *d*_out_ would then be 0.93*R*. Conversely a random orientation for the telomere cap would give a distribution with an average of 1.31*R*. The data (combining *Ph1*+ and *Ph1*− cells; Figure 4D), with average 1.30*R*, matches much better with this second case, showing that the initial cap containing the telomeres does not have a preferred orientation. This result is unchanged if we separately analyse *Ph1*+ and *Ph1*− cells. This conclusion is important for our mathematical model, as we explain below.

Although the average of *d*_out_ matches well between the experimental data and our cap distribution, the full experimental distribution is still somewhat broader (Figure 4D). Nevertheless, the width of the distribution for a randomly-orientated cap is much closer to the experimental value than that for an “outside” cap (see Supporting Information).

The Rabl configuration may also influence the position of telomere clusters within the randomly-orientated cap. For example, chromosomes with similar arm lengths may tend to lie closer to each other than to other chromosomes. This may help explain the spread in initial cluster size, with some sister chromatid telomeres grouped in pairs and others grouped in larger clusters. In principle it would be possible to explicitly model the position of the full length of each chromosome, via a semiflexible polymer model (as in [20] and [12]), in order to determine the initial telomere positions. However, such an analysis is beyond the scope of this work and instead we assume, for simplicity, that the telomere clusters are initially placed randomly within the randomly-orientated cap.

### Bouquet formation is delayed in meiocytes in the presence of the *Ph1* locus

Since the *Ph1* locus has been implicated in preventing homeologous pairing, it may well affect the dynamics of telomere bouquet formation. To study this issue we considered telomere data both from a wheat-rye hybrid with the *Ph1* locus (*Ph1*+) and in a mutant where the locus has been deleted (*Ph1*−). As expected, we found a wide range in the number of telomere clusters in the *Ph1*− meiocyte images, ranging from many separate clusters to a single large cluster after the bouquet has formed (see below). However, interestingly, when we plotted the histogram of the number of telomere clusters for *Ph1*+ meiocytes (Figure 4B), we found little evidence of bouquet formation. In fact we found a distribution that is remarkably similar to the distribution for tapetal cells (Figure 4A). The bouquet eventually forms even in *Ph1*+ meiocytes [16], but since our images (from around the time of meiocyte replication) do not show any noticeable change from the tapetal telomere distribution, we conclude that the onset of bouquet formation is delayed in the presence of *Ph1*. Since it is not clear whether homologous pairs form before or after formation of the bouquet, perhaps the purpose of this delay in the presence of *Ph1* is to facilitate correct pairing of homologues, allowing more time to check potential pairings and dissociate incorrectly-paired homeologous chromosomes.

To further confirm our conclusion that bouquet formation has apparently not started in our *Ph1*+ meiocyte dataset, we also compared the *d*_max_ and *d*_out_ distributions from *Ph1*+ meiocytes with those from the tapetal (both *Ph1*+ and *Ph1*−) data. Histograms for *d*_max_ and *d*_out_ (Figure S2) are, as with tapetal cells, consistent with telomere confinement to a randomly-orientated cap, whose opening angle is slightly less than that of a hemisphere (∼170° on average). Further the average number of telomere clusters is 26±1, again agreeing with the tapetal data (the error is the standard error in the mean). The width of the cluster number histogram, 9, is similar again to that for tapetal cells. The fact that both tapetal cells (with and without *Ph1*) and *Ph1*+ meiocytes share the same telomere cluster distribution, both in terms of number and position, buttresses our hypothesis that *Ph1*− telomeres also start in the same configuration.

### *Ph1*− meiocytes show telomere bouquet formation dynamics

**Figure 5:**
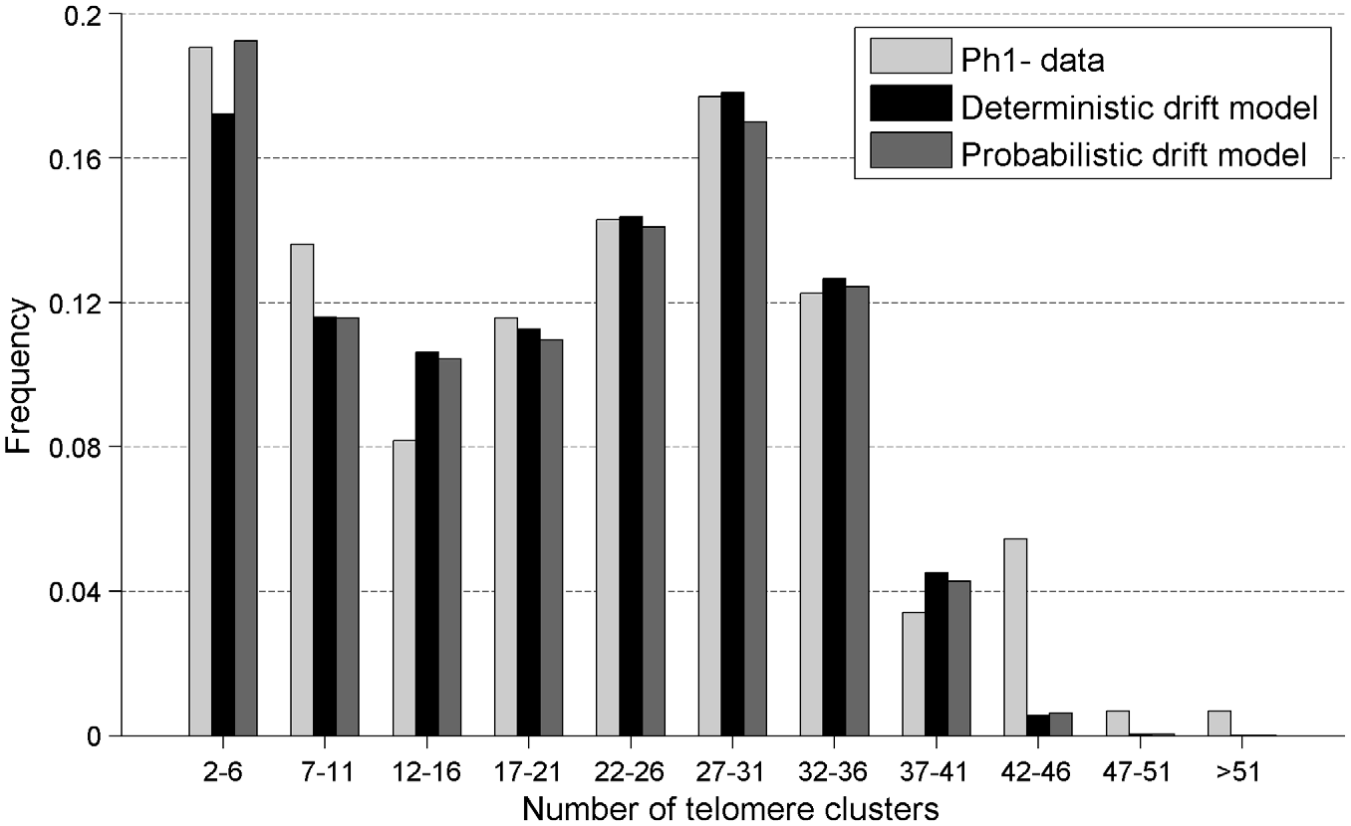
Distribution of the number of telomere clusters in *Ph1*− meiocytes, showing both the experimental data (n = 159) and a fit to the pure deterministic drift model in which telomeres move directly to the outside pole from an initially randomly-orientated cap. Also shown is the best fit for the probabilistic drift model where the drift direction is itself drawn from a distribution (with standard deviation δψ = 40° and run length *L*_R_ = 1 µm).

In contrast, *Ph1*− meiocytes do exhibit intricate bouquet-forming telomere dynamics. This is seen in the histogram of the number of telomere clusters (Figure 5), which now has a second peak at the origin, representing nuclei that are close to completing bouquet formation, with only a few remaining clusters. Although the dynamics of bouquet formation is clearly visible in the histogram for small numbers of clusters, the large peak around 28 clusters seen in *Ph1*+ meiocytes and tapetal cells (in both *Ph1*+ and *Ph1*−) is still visible, supporting the idea that telomere clusters in *Ph1*− meiocytes also start in the same configuration. We believe the presence of two peaks is due to our imaging dataset capturing not only the dynamic formation of the bouquet, but also the period before bouquet-formation onset, when the telomere clusters are arranged in their initial configuration. This leads to the superposition of the initial telomere cluster distribution and the bouquet dynamics distribution (as seen in Figure 5).

### Meiocytes within the same floret initiate bouquet formation at similar times

It is an interesting question as to how multicellular organisms coordinate meiosis amongst their constituent cells. In wheat, for example, meiosis within different meiocytes could be coordinated at various levels, including that of single meiocytes, locules, anthers, florets or even spikelets (see Figure 1A). Our *Ph1*− meiocyte data originates from anthers within 12 separate florets and so we were also able to study whether individual florets showed synchrony in the time that bouquet formation started, i.e. whether all meiocytes in a given floret begin to form the bouquet at the same time. In fact it may be that the synchrony is not within florets, but at a lower level, say within anthers. However, since our dataset is only split into florets, we can only test synchrony within florets. To investigate this question we split our data into separate florets and considered the cluster number distribution for each. If florets are synchronous then we would expect a tighter distribution, i.e. smaller variance, for individual florets compared to that for all the florets combined. We found the mean variance in the number of clusters for individual florets was only 66±18 (this error is the standard error in the mean variance), compared to 160 for the whole data set (see Supporting Information). To test whether this was significant we repeatedly randomly partitioned the complete dataset into 12 appropriately-sized sub-datasets, finding that the mean variance followed a distribution with mean 160 and standard deviation 18 (see Supporting Information). Since a mean variance of 66 is far from the randomly-partitioned mean variance of 160, we conclude that individual florets do indeed show synchrony, with meiocytes within many florets starting bouquet formation at similar times.

However, it is worth noting that the distribution of the variances in cluster number for individual florets, with mean 66, has a relatively large standard deviation of 61, which suggests that, although many florets show synchrony, this may not be true of all florets. This may be because the synchrony is not within florets, but within anthers. It would be interesting to test whether synchrony is only at the anther level by studying images from individual anthers.

### Mathematical modelling of bouquet formation

To understand better the mechanics of telomere bouquet formation, we constructed a mathematical model incorporating the dynamics of telomere clusters moving along the nuclear membrane. We then used the model to simulate bouquet formation. To do this we idealised the nuclear membrane as a sphere of radius *R*, with each telomere cluster represented by a position on the surface of the sphere. Although nuclei are never exactly spherical, they are frequently close to this ideal (with the centre-to-edge distance, on average, varying by only about 10%) and so we do not expect a spherical approximation to substantially affect our results.

#### Initial conditions

We began each simulation with *n_0_* telomere clusters confined to the membrane, where *n_0_* was chosen from a Gaussian distribution. The average, <*n_0_*>, and width, δ*n_0_*, for this Gaussian are similar to those for the telomere cluster distribution in tapetal cells (Figure 4A), although, in fact, a slightly better fit to the *Ph1*− data was obtained by using a mean and standard deviation of 32 and 6 respectively, rather than 27 and 8 as found for tapetal cells and 26 and 9 as found in *Ph1+* meiocytes. This suggests that the initial telomere clusters in *Ph1−* meiocytes may not be as tightly bound as in *Ph1+* meiocytes, although we do not have enough data to prove this conclusively. First we choose the cap that initially contains the telomere clusters, which involves picking a random opening angle for the cap (from a Gaussian distribution with mean 170° and standard deviation 30°) and picking an orientation for this cap (by choosing an angle from a uniform distribution on the sphere surface). Then telomere clusters are placed on the sphere surface, at random positions within the cap. In this way the initial telomere cluster positions in the simulations closely matched the initial telomere cluster distributions measured experimentally. Before the telomere clusters were allowed to move, we implemented a waiting time, *T*_0_, where there are no telomere dynamics. This captures the fact that, in addition to telomere dynamics, our images also include earlier times before the meiocytes begin bouquet formation. In addition we chose a random position for the bouquet site from the distribution given in Figure S1. This corresponds to our observation that the bouquet site is not always directly opposite the anther centre.

#### Implementation of telomere cluster dynamics

At each time step (Δ*t* = 25 s), we allowed telomere clusters to move along the membrane in two ways: drift and diffusion. For theoretical work describing this process for a single telomere see [21]. Drift, which is directed movement in a specific direction (in this case along a great circle towards the bouquet site), is presumed to be due to motors moving chromosomes along some component of the cytoskeleton (perhaps dynein walking along microtubules, with the link to telomeres provided by SUN-KASH proteins). For simplicity we assumed that telomeres always drift with constant speed, *v*, and so, at each time step, each telomere cluster moved along a great circle a distance *v*Δ*t* directly towards the bouquet site. Randomly directed diffusive motion could be thermal, due to Brownian motion of individual chromosomes, but could also be due to active (perhaps motor-driven) random motion (potentially due to a completely disordered cytoskeleton). To implement diffusion with diffusion constant *D*, telomere cluster motion was split into two orthogonal directions (since telomeres are confined to the membrane, diffusion is two-dimensional). For each direction there was an independent probability (*p* = *D*Δ*t*/(Δ*x*)^2^) of taking a step forward (of distance Δ*x*) and an equal probability of taking a step backwards (also of distance Δ*x*), where Δ*x* = 0.1 µm. We checked that our values of Δ*t*, Δ*x* and *p* were sufficiently small to correctly implement diffusion. See Supporting Information for more details.

When telomere clusters were within a certain distance λ (called the bouquet radius) of the pole, they were considered to be part of the bouquet and no longer underwent dynamics. We normally used λ = 3.6 µm, although we checked that changing λ did not qualitatively change our conclusions (see Supporting Information). The simulation ended when all telomere clusters were part of the bouquet, i.e. when there was only one cluster. For the parameters used in the simulation, see Table 1. To match with our experimental data we recorded the number of telomere clusters plus one (for the bouquet itself) at randomly chosen time points. These time points were chosen such that each second there was a 10^−5^ probability of recording data, which resulted in less than 1 reading per simulation, similar to our experimental data, which consists of individual snapshots of telomere positions. We also recorded the total time for bouquet formation. Finally, at each time step we measured both *d*_max_ and *d*_out_.

**Table 1:** Parameter values used in the simulations.

Parameter	Symbol	Value
Nuclear membrane radius	*R*	8 µm
Mean initial number of telomeres	*<n_0_>*	32
St. dev. of initial number of telomeres	*δn_0_*	6
Drift speed	*v*	8.5×10^−4^ µms^−1^
Diffusion constant	*D*	various; see text
Waiting time	*T*_0_	72 mins
Bouquet radius	λ	3.6 µm
Mean initial cap opening angle	<Θ_0_>	170°
St. dev. of initial cap opening angle	δΘ_0_	30°
Standard deviation of drift direction	δψ	various; see text
Run length	*L*_R_	1 µm

We also considered various extensions to our model. Firstly we included telomere clusters that bind when they approach within a certain radius. In this model two telomere clusters within some fixed distance are replaced by one telomere cluster at their centre of mass (the “mass” of a cluster is proportional to the number of telomeres it contains). We also considered the case that telomere clusters had a non-zero size and so could not approach each other too closely. To implement this effect all telomere clusters were modelled as hard spheres whose radii were proportional to the number of telomeres in the cluster. Clusters that tried to drift or diffuse within this radius were forbidden from doing so. Finally, we considered clusters whose diffusion constant and drift speed depended on how many telomeres they contained. We achieved this by allowing the drift speed and diffusion constant of each cluster to be inversely proportional to the number of telomeres in the cluster. See Supporting Information for details of these extensions. However, none of the extensions led to any noticeably improved matching with the experimental cluster number histogram.

#### Parameterisation

Although our experimental setup cannot directly measure the total time for bouquet formation, *T*_bouq_, this was measured in [14] for rye, where it was found that *T*_bouq_ = 6.3±0.5 hr, i.e. a variation in the time for bouquet formation of about 8%. We assume that this value will be similar in our wheat-rye hybrid.

Although we are unable to directly measure the waiting time, *T*_0_, we can infer its value by fitting to the cluster number histogram. The effect of increasing *T*_0_ is to increase the size of the peak around *N* = 27–31 in Figure 5, and so by fitting the ratio of the peak at *N* = 27–31 to the peak in the first bin (*N* = 2–6), we can determine *T*_0_. Similarly we fit the two parameters controlling the initial number of telomere clusters, <*n_0_*> and δ*n_0_*, by ensuring the peak at around *N* = 27–31 has the correct mean and standard deviation respectively.

Apart from a parameter that we directly measure (*R*), the parameters that are fit to the cluster number histogram (*T*_0_, <*n_0_*> and δ*n_0_*), the parameters we fit to the meiocyte *Ph1*+ and tapetal *d*_max_ and *d*_out_ histograms (<Θ_0_> and δΘ_0_), and a parameter that only mildly affects the dynamics (λ; see Supporting Information), our model contains only two adjustable parameters: the drift speed, *v*, and the diffusion constant, *D*.

We consider distributions for both the cap opening angle, Θ_0_, and for the initial number of telomere clusters, *n*_0_, and fix all other parameters (Table 1). We could also consider fluctuations in the other parameters, such as the drift speed, *v*, the membrane radius, *R*, and the waiting time, *T*_0_. Interestingly, however, such additional variation (with up to nearly 50% relative variation in the drift speed) did not lead to any better fit with the experimental data (see Supporting Information).

### A pure directed movement model can explain bouquet formation dynamics

First, we considered a pure diffusional model, with no drift. Although this can lead to bouquet formation in the correct total time it is unable to capture only an 8% variation in this time. In fact, with *D* = 0.025 µm^2^ s^−1^, we found *T*_bouq_ = 6.6±2.6 hr, with an approximately 40% variation. Further, such a model cannot capture the cluster number histogram shown in Figure 5, since the minimum around *N* = 12–16 is too pronounced. Thus a pure diffusion model is unlikely, which agrees with the same conclusion in [14].

**Figure 6:**
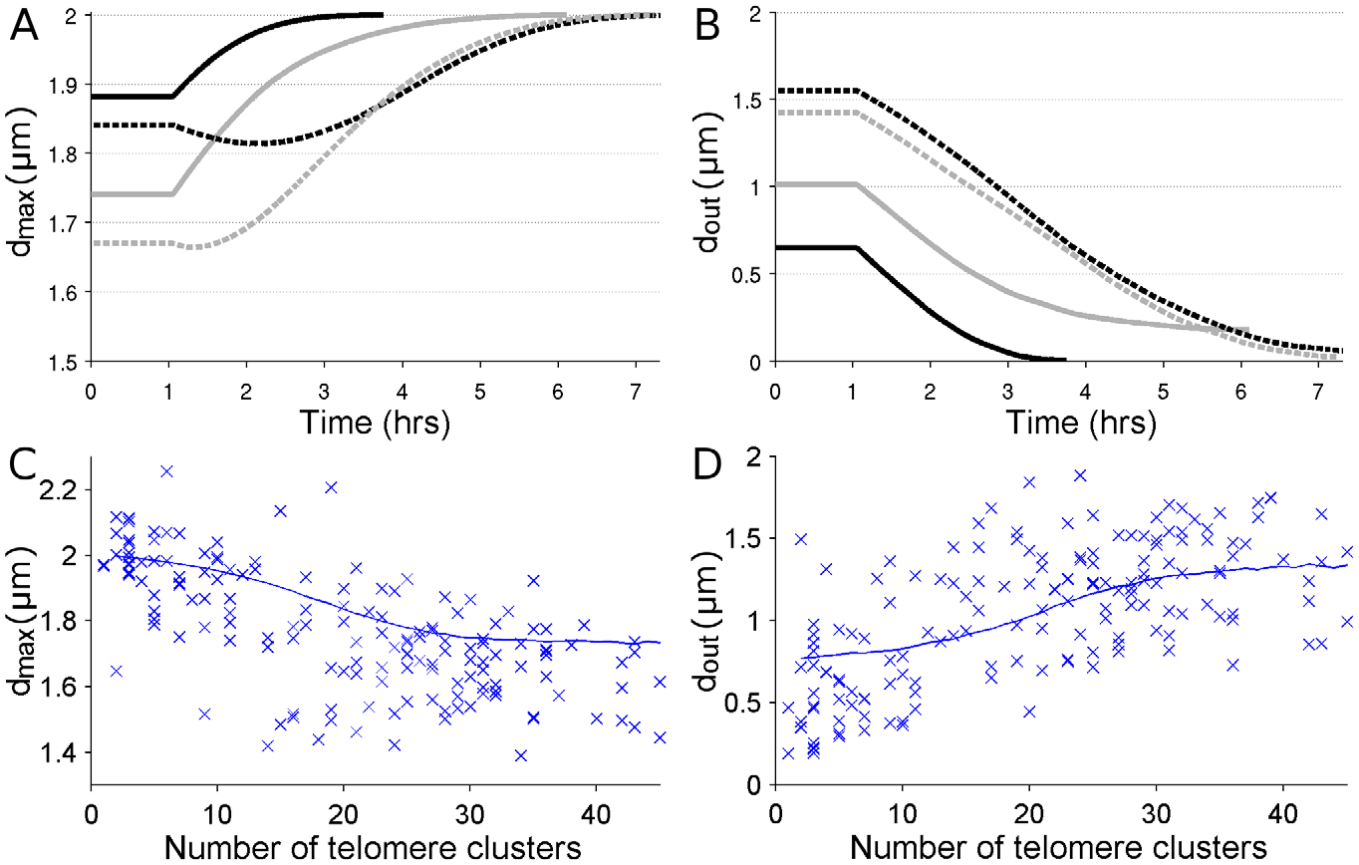
Examples of how *d*_max_ and *d*_out_ change with time, and experimental scatter plots (compared to the deterministic pure-drift model) of how *d*_max_ and *d*_out_ vary with the number of telomere clusters. A. Four examples of how *d*_max_ changes with time in the deterministic pure drift model with a randomly-orientated initial cap. As telomere clusters move towards the bouquet site, *d*_max_ generally increases attaining a maximum of 2*R* when the bouquet is fully formed. The initial plateau represents the initial waiting time *T*_0_. Cases where *d*_max_ initially decreases correspond to the initial cap partially occurring in the inside hemisphere (i.e. the hemisphere opposite the bouquet site). B. The same four examples but showing how *d*_out_ changes with time. In contrast to *d*_max_, *d*_out_ decreases as the bouquet forms, reaching a minimum upon bouquet completion. The theoretical minimum (*d*_out_ = 0) is only attained if the bouquet site is directly opposite the centre of the anther. C. Maximum average telomere cluster distance (as a fraction of the nuclear radius), *d*_max_, against the number of telomere clusters, *N*, for *Ph1*− meiocytes. D. Average telomere cluster distance to outside pole (as a fraction of the nuclear radius), *d*_out_, against the number of telomere clusters, *N*, for *Ph1*− meiocytes. The blue lines show the average values of *d*_out_ and *d*_max_ given by the deterministic pure-drift model.

Next we considered a pure drift model, without any diffusion. In [14] this was excluded since it could not match the observed variation in *T*_bouq_, producing far too small a variation. However, our initial conditions are different: our telomeres are initially contained in a randomly-orientated cap, rather than in an outwardly-pointing hemisphere as in [14]. This makes a crucial difference since now the dominant source of variation in *T*_bouq_ is due to the initial cap orientation. With *v* = 8.5×10^−4^ µms^−1^ we found that *T*_bouq_ = 5.6±1.0 hr. This is in good, although not perfect, agreement with the observed value in [14] (although we note that the standard deviation in [14] is for rye and is based on only four measurements). Thus, by appreciating that the initial cap containing the telomeres is not necessarily on the outside of the anther, a pure drift model can explain the data. Figures 6A and 6B shows examples of the evolution of *d*_max_ and *d*_out_ for the pure drift model.

Further, the pure drift model appears to match well with the cluster number histogram (Figure 5). To test this more rigorously we performed a chi-squared goodness of fit test, which gave a test statistic of 2.1, a value that is well below that required to doubt the model at the 5% confidence limit (which is 9.5). Thus there is no reason to reject the null hypothesis that our experimental data is well-described by our pure drift model. See Supporting Information for more details. This good match between model and data would not have been the case if the telomere clusters had started in the outside hemisphere rather than a randomly-orientated cap. In this latter case (as in the experimentally measured data) the histogram contains an extra peak at small numbers of clusters that is caused by the few telomere clusters that start on the side of the nucleus opposite to the bouquet and take a relatively long time to move towards the bouquet site. It is these final few telomere clusters that are the last to join the bouquet and, since they are few in number (due to the decreasing area near the poles), there are relatively long gaps between the final few telomere clusters joining the bouquet, leading to the extra peak for small cluster numbers. We also considered the behaviour of *d*_max_ and *d*_out_ as a function of the number of telomere clusters for *Ph1*− meiocytes. Despite the noise in the data, the pure drift model still captures the overall telomere dynamics (Figures 6C and 6D).

Another way of quantifying telomere dynamics is via the average telomere cluster distance, *d*_pairs_, which is defined as the mean distance between all pairs of telomere clusters (without intensity-weighting). As the bouquet forms and all telomeres approach a point close to the outside pole, this distance tends to zero. If we plot *d*_pairs_ as a function of time for our pure drift model we find two categories of behaviour (Figure S5). Firstly, for cases where the initial cap is mostly facing the outside of the nucleus (nearest where the bouquet forms), *d*_pairs_ is monotonically decreasing. Secondly, however, there are cases where the initial cap is partially facing inwards, where we find that *d*_pairs_ initially rises before eventually dropping to zero. This initial rise is due to telomere clusters that start near the inside pole of the nucleus and must first diverge from each other as they proceed through the equator, until they form the bouquet near the outside pole. When we average over all initial cap orientations we find, after the initial period of *T*_0_, a slight rise in *d*_pairs_, followed by a relatively gentle decrease, before *d*_pairs_ finally drops to zero (Figure S5). This effect was also noted in [14], where its origin was mysterious, and was suggested to be due to a short period between relaxation of the Rabl configuration and directed movement towards the bouquet site, when telomere clusters were able to diffuse freely. Our model, however, provides a natural explanation for this behaviour without the need to postulate an extra period of free diffusion before bouquet formation starts.

As we have seen, a pure directed movement model can fit our data, and hence there is no necessity for diffusive motion. The effect of adding such diffusion is to increase the peak at small cluster numbers (since the final few telomere clusters take a relatively long time to find the bouquet due to partially random rather than directed movement). This effect can be compensated for by increasing the waiting time, *T*_0_, although then the trough at *N* = 12–16 is more pronounced. Small amounts of diffusion still match the experimental data and so cannot be excluded. However, the important point is that diffusion is not required in our model to fit our data and since, as we discuss below, global diffusion may be negligible, we predict that pure drift is the relevant mechanism in our system.

### Incorporation of a probabilistic drift direction demonstrates robustness to cytoskeletal disorder

In the simplest version of the model, telomere clusters move with constant drift speed directly towards the bouquet site. Although we do not know which component of the cytoskeleton is responsible for causing this drift, such a model would require a perfectly ordered cytoskeleton, with all cytoskeletal elements near the nucleus pointing directly towards the bouquet site. Although there is some evidence that microtubules in plants lie tangential to the nucleus during prophase [22], it is not clear to what extent there is variation within this arrangement. In fact, in rye there is evidence that, during bouquet formation, there is significant disorder to the microtubules near the edge of the nucleus [23], with perhaps only the overall average direction pointing towards the bouquet site.

To model this, we introduce a random element to the drift direction in the model. Unless the drift direction varies enormously, we emphasise that this is not the same as simply adding diffusion since telomeres still almost always head roughly towards the bouquet site. Rather this situation is more like constant drift directed towards the bouquet site, with a small amount of transverse diffusion. Telomere clusters are assumed to drift in some direction (not necessarily directly towards the bouquet site) for some distance, *L*_R_, called the run length. Without knowing the details of the cytoskeletal elements involved in telomere movement, it is difficult to estimate the run length. However, motivated by studies of kinesin [24], we use *L*_R_ = 1 µm. The drift direction, ψ, is chosen (independently for each telomere cluster) from a truncated normal distribution, with mean centred on the direction towards the bouquet site and with standard deviation, δψ. After a telomere cluster has moved the run length in this drift direction, a new independent drift direction is chosen. The standard deviation of the drift direction, δψ, is thus a measure of the directionality of the cytoskeleton. See Supporting Information for more details. It is worth noting that, even with a random element to the drift direction, a randomly-orientated initial cap is still required to produce a good match with the experimental cluster number histogram.

To fit with the experimental data it is necessary, for each value of δψ, to refit both the drift speed, *v*, and the waiting time, *T*_0_. After doing this it is notable that a non-zero variation in the drift direction can produce a slightly better match with the experimental cluster number histogram (Figure 5). To quantify this, we use the chi-squared test-statistic as a measure of the goodness-of-fit. As δψ increases from zero the value of the statistic decreases, reaching a minimum of 1.6 at around δψ = 40°, with similar results obtained if the run length is reduced to *L*_R_ = 0.5 µm. So although we do not know the relevant run length for telomere movement, for reasonable values we predict that even a relatively large variation in the cytoskeletal directionality does not seem to interfere with bouquet formation.

## Discussion

The arrangement of chromosomes within the nucleus is far from random, with radically different arrangements required during interphase, mitosis and meiosis. Even during interphase there is significant chromosomal order, with ribosomal DNA localising to the nucleolus, actively transcribed genes often present in transcription factories, and discrete chromosome territories for individual chromosomes. Further, during mitosis sister chromatids must be segregated, which involves kinetochores forming at the centromeres and being pulled apart by microtubules to opposite ends of the cell. A different arrangement again is required during meiosis, with telomeres often grouped together on the nuclear membrane and homologous chromosomes forming bound pairs, often accompanied by complex motions of the entire chromatin [9].

Although the process of chromosome segregation during mitosis is well-studied, much less is known about how the telomere bouquet forms during meiosis. In fact the bouquet itself is not a universal feature in all organisms. For example, *Arabidopsis thaliana* lacks a bouquet, with the telomeres instead associating with the nucleolus [25]. Further, some organisms do not even use the telomeres to facilitate pairing. *C. elegans*, for example, attaches special pairing centres to the nuclear membrane rather than telomeres [26]. In fact, organisms seem to display substantial variation in exactly how chromosomes are organised during meiosis.

Despite this variation, in general terms, almost all organisms associate specific regions of the chromosomes (telomeres or pairing centres) to some relatively small region of the nucleus (nuclear envelope or nucleolus) and often use cytoskeletal elements to move chromosomes around this region, sometimes gathering all telomeres in an even smaller bouquet region. Again, there is variation as to which component of the cytoskeleton is used, with animals and fission yeast tending to use microtubules and budding yeast instead employing actin. For example, it seems that dynein motors are used by *C. elegans* to move the pairing centres along microtubules [27], [28].

From a meiotic viewpoint, wheat is in many ways an extreme case. Partly due to being hexaploid and partly due to many repetitive DNA elements, the wheat genome is over a thousand times larger than that of yeast. Necessarily this then implies a large nucleus and in fact the wheat nucleus has several hundred times the volume of a typical yeast nucleus. Thus with very large (and hence potentially slow moving) chromosomes needing to search relatively large nuclei to find their homologous partner, the problem of homologous pairing is much more pronounced in wheat than in many other organisms. Further, the problem of partner identification is compounded by the presence of related but non-homologous (i.e. homeologous) chromosomes and large amounts of repeated DNA. So perhaps it is not surprising that, as meiosis progresses and the telomere bouquet is almost formed, centromeres in wheat associate into homeologous clusters, such that seven centromere groups can be seen per nucleus [15]. This centromere clustering, which is in addition to the telomere clustering modelled in this paper, is another level of chromosome organisation that wheat employs, perhaps to overcome problems related to the large chromosome and nuclear size.

In addition to this grouping of centromeres shortly before bouquet formation is complete, we have shown that even prior to the initiation of bouquet formation, after telomeres have moved to the nuclear envelope, wheat also groups sister chromatid telomeres together. Interestingly, this occurs not only in meiocytes, but also in tapetal cells, which at no point undergo meiosis. Unlike centromere clusters, where each of the seven centromere groups is likely to be composed of six homeologous chromosomes, sister chromatid telomeres tend to cluster, on average, in pairs, so that each cluster contains, on average, four telomeres. Such initial telomere association has also been observed in other organisms, such as rye [14] and a maize mutant [29]. Since wheat-rye contains only homeologous rather than homologous chromosomes, the sister chromatid telomeres cannot be homologous pairs. Given this, it would be interesting to determine whether the association is between sister chromatid telomeres at opposite ends of a chromatid pair, between homeologous chromosomes or whether any set of sister chromatid telomeres can cluster together.

Once on the membrane, the telomere clusters move together to form the bouquet. Interestingly, this bouquet normally forms at the pole of the nucleus furthest from the centre of the anther. A similar result was found in rye [23]. Pure diffusion is not sufficient to explain the variation in the bouquet formation time and our model confirms that directed movement is also required, agreeing with the result in [14]. As such we predict that there must be significant organisation to the cytoskeleton (or some other similar structure), forming a directed network along which telomeres can move towards the bouquet patch. Since no SUN/KASH proteins linking telomeres through the nuclear membrane have yet been discovered in wheat, it is unclear which structural elements are involved. Possibilities include microtubules (as in animals), actin (as in *S. cerevisiae*), nuclear envelope structural proteins (similar to the nuclear lamins in animals), or perhaps even the controversial idea of a nuclear matrix. Potentially relevant is the fact that the process in rye has been shown not to involve microtubules [30].

Although a mixture of diffusion and directed movement can form the bouquet, we have shown that diffusion is not required. Our data can be explained by a pure drift model, with telomere clusters moving directly towards the bouquet site near the outside pole. Although there must always be some diffusion, we believe that such diffusion is unlikely to play any significant role in forming the bouquet. This is because chromosomes are effectively confined to small regions potentially due to the barriers formed by other chromosomes. Chromosomes do diffuse, but only within these small regions [31], such that diffusion is unlikely to be relevant in moving telomere clusters all the way to the bouquet site. For example, during interphase in *S. cerevisiae* it has been shown that chromosomes freely diffuse, but only within a region of radius 0.3 µm, which is considerably smaller than the radius of the yeast nucleus [32]. Similarly, results in *Drosophila* suggest that chromosome regions are confined within a 0.5 µm radius [33]. Thus any diffusional component may well be irrelevant, further supporting our pure drift model.

Although we favour a pure drift model, this does not imply that the telomeres need always drift directly towards the bouquet site. In fact, a model where the drift direction varies, with a relatively large standard deviation of around 40° fits the data slightly better. This model is similar to one with constant drift towards the site of bouquet formation together with transverse diffusion (as distinct from diffusion in any direction discussed above). Interestingly, this version of the model predicts that bouquet formation dynamics is robust to significant cytoskeletal disorder. This issue could potentially be investigated in the future by imaging the relevant cytoskeletal component(s).

The *Ph1* locus seems to have evolved in wheat in order to ensure that only homologous chromosomes pair during meiosis. Since wheat originated from the hybridisation of three related diploid species, each chromosome occurs in six similar copies, and so pairing must be carefully controlled to restrict the formation of homeologous pairs. If the *Ph1* locus is deleted then both homologous and homeologous pairs form, which eventually leads to infertility. Our analysis has shown that *Ph1*− mutants complete telomere bouquet formation at earlier times. Reduced Cdk2 activity during mammalian meiosis affects telomere behaviour and their attachment to the nuclear membrane [34]. The presence of the *Ph1* locus has recently been shown to reduce Cdk2-type activity in wheat-rye hybrids [16]. Thus the modified telomere behaviour with and without *Ph1* observed here is entirely consistent with altered Cdk2-type activity.

Formation of the telomere bouquet is an important step during meiosis in many organisms [2], [35] and the relative roles played by drift and diffusion in this process is an important question. We have shown that the arrangement of telomere clusters prior to bouquet formation is of vital importance for understanding later telomere dynamics, with telomere clusters initially positioned in a randomly-orientated cap whose size is slightly smaller than that of a hemisphere. We have argued that a pure drift model, which is probably the simplest possible mechanism, may well be the most appropriate. This is distinct from previous models where a diffusional component was required. Although our results were obtained in a wheat-rye hybrid, the confined nature of diffusion suggests that similar mechanisms may be used in many organisms, and it is quite possible that the formation of most telomere bouquets is best understood as due to purely directed movement.

## Materials and Methods

### Plant materials

The plants used came from crosses between rye (*Secale cereale* cv. Petkus) and hexaploid wheat (*Triticum aestivum* cv. Chinese Spring). Two crosses were used: one from wild-type wheat and one with the wheat lacking the *Ph1* locus [36]. Seeds from both genotypes were germinated on petri dishes for 3–4 days. The seedlings were vernalized for 3 weeks at 4°C and then transferred to a controlled environmental room with the following growth conditions: 16 hours at 20°C (day) and 8 hours at 15°C (night) with 85% humidity. Plants were collected after 6–7 weeks.

### EdU treatment

EdU treatment was carried out as described previously [16]. Briefly, tillers containing immature pre-meiotic spikes were detached after an 8 hr period in the dark and immediately transferred to a solution of 100 mM sucrose and 1 mM EdU (Invitrogen: A10044). The cut tillers in individual tubes were left in the light for four hours, after which the spike was dissected out and fixed in 4% formaldehyde solution, freshly made from paraformaldehyde [37]. The fixed samples were placed in biopsy cassettes and embedded in wax using a Tissue-tek vacuum infiltrator processor (VIP) machine [38]. They were then sectioned using a microtome [25] to produce sections of 10 µm thickness.

### EdU detection and fluorescence in situ hybridization

EdU detection was carried out using a Click-iT EdU Alexa Fluor 488 Imaging Kit, according to the manufacturer's instructions (Invitrogen: C10337).

Fluorescence in situ hybridization was carried out as previously described [39]. Telomeric probes were labelled with biotin-16-dUTP by nick translation of PCR-amplified products using the oligomer primers (5′-TTTAGGG-3′)_5_ and (5′-CCCTAAA-3′)_5_ in the absence of template DNA [40], and detected using streptavidin-Cy3 conjugate. Chromosomes were counterstained with DAPI (4′,6-diamidino-2-phenylindole) and mounted in Vectashield (H-1000) medium.

To carry out dual labelling with both EdU and telomere probes, samples (sections) were first digested in 2% cellulose, 2% pectolyase in 1×TBS for 3 hours at 37°C. Then, the telomere probe was hybridised to the sample, incubated overnight at 37°C and washed according to the usual protocol [39]. Samples were then blocked in 3% BSA and followed by the EdU detection protocol. Before staining with DAPI, samples were incubated with the required antibodies for the telomere probe, and then finally mounted in Vectashield.

### Image acquisition and analysis

Images were acquired with a Nikon Eclipse E600 epifluorescence microscope equipped with a Hamamatsu Orca-ER cooled CCD camera and a Prior Proscan x,y,z stage. Stack images of individual cells were collected by using MetaMorph (Universal Imaging) software. Deconvolutions of images were processed with AutoDeblur (AutoQuant Imaging). Projections of 3D pictures were performed with ImageJ. These images were also used in [16] to study the possible Cdk2-type activity of the *Ph1* locus. However, the work described here is the first detailed analysis of this data, where the actual telomere cluster positions are determined and analysed.

From each DAPI image we first identified the nucleus by generating ellipses that fit around the DAPI stain. Then, from the equivalent telomere FISH image, we identified telomeres within the nucleus by searching for discrete pixels with an elevated FISH signal. Pixels were only counted as part of a telomere if their intensity was greater than some threshold, which, for each image, was chosen as 0.12 of the maximum pixel intensity. This value was chosen since it gave consistently-sized clusters: larger values missed some telomere clusters and smaller values sometimes led to large regions being incorrectly identified as a single cluster. After imposing the threshold, clusters were defined such that adjacent pixels (including diagonally adjacent) were considered part of the same cluster. In order to remove spurious background signals, clusters were only counted if their total intensity was greater than 2 (where each pixel had a maximum intensity of 1). We tried various other values for the minimum total intensity, although this did not affect our results. See Supporting Information for more details.

## Supporting Information

Figure S1 Histogram of the angle between the telomere bouquet and the “outside” pole of the nucleus (measured from the centre of the nucleus) for *Ph1*− meiocytes near to or after completion of the bouquet (n = 35).
(TIF)

Figure S2 Histograms quantifying the telomere cluster spatial distribution in our *Ph1*+ meiocyte dataset (n = 74), compared to the theoretical situation where telomere clusters lie randomly positioned in a randomly-orientated cap subtending an opening angle of 170°±30°. A. Distribution of maximum average telomere cluster distance, *d*_max_ (as a fraction of the nuclear radius). B. Distribution of average telomere cluster distance to outside pole, *d*_out_ (as a fraction of the nuclear radius).
(TIF)

Figure S3 Comparison of the telomere cluster number distribution for two versions of the deterministic pure drift model with a randomly-orientated initial cap: one with λ = 0.5 µm, *T*_0_ = 1 hr and one with λ = 6 µm, *T*_0_ = 3 hr.
(TIF)

Figure S4 Average total time for bouquet formation against the diffusion constant for a constant drift speed of 8.5×10^−4^ µms^−1^. The error bars are too small to be visible.
(TIF)

Figure S5 Behaviour in time of the average telomere cluster distance, *d*_pairs_, in the deterministic pure drift model with a randomly-orientated initial cap. Blue lines: individual examples. Red line: average over many initial conditions.
(TIF)

Text S1 Important additional information related to the image analysis, the data analysis and the computational modelling.
(DOC)
