# Supplementary figures and images for: Quantitative Dynamics of Telomere Bouquet Formation
Source: PLoS Comput Biol. 2012 Dec 6;8(12):e1002812. doi: 10.1371/journal.pcbi.1002812 (PMC3516562; doi:10.1371/journal.pcbi.1002812)

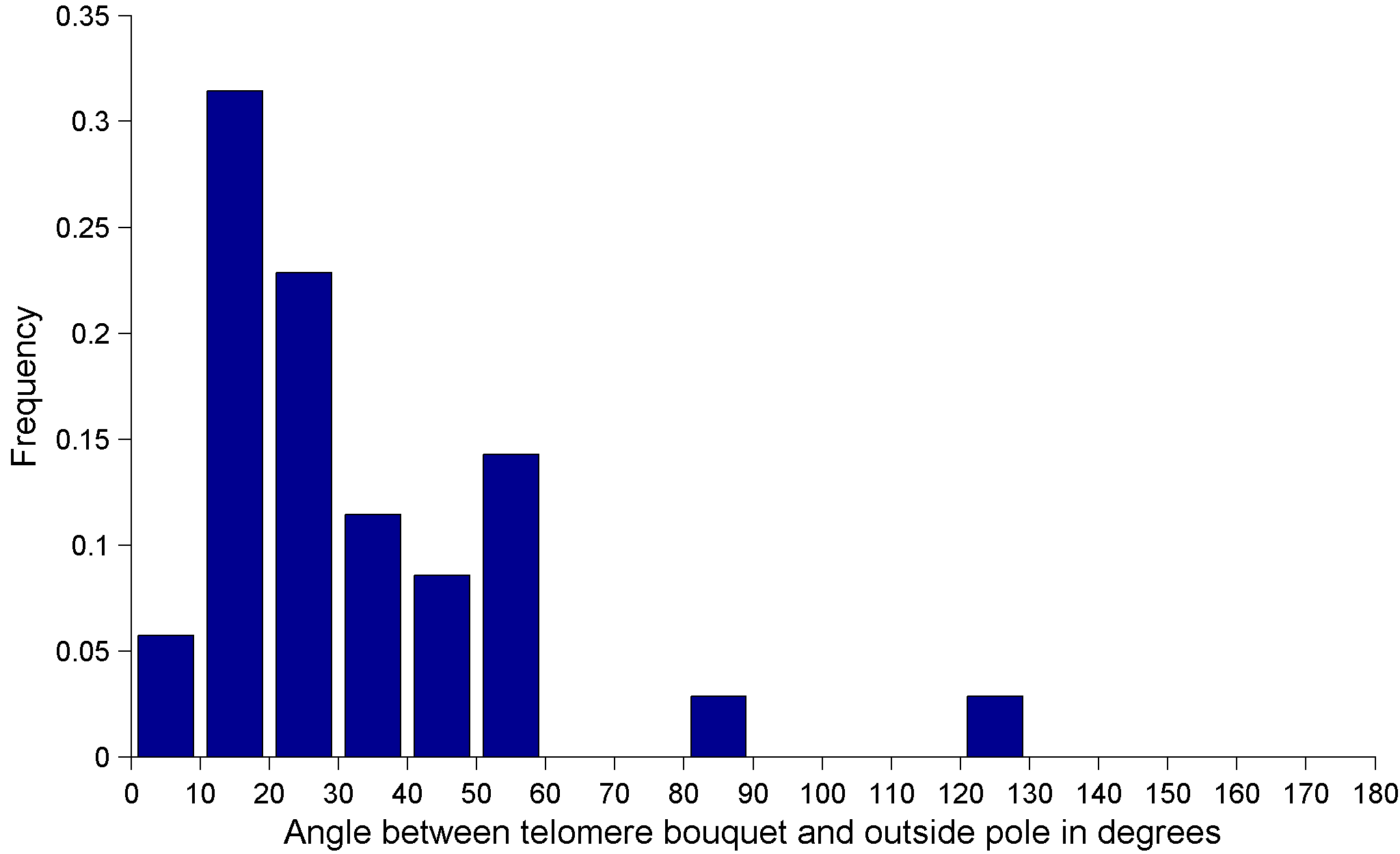

Supplement: Figure S1 — Histogram of the angle between the telomere bouquet and the “outside” pole of the nucleus (measured from the centre of the nucleus) for Ph1− meiocytes near to or after completion of the bouquet (n = 35). (TIF) [file pcbi.1002812.s001.tif]

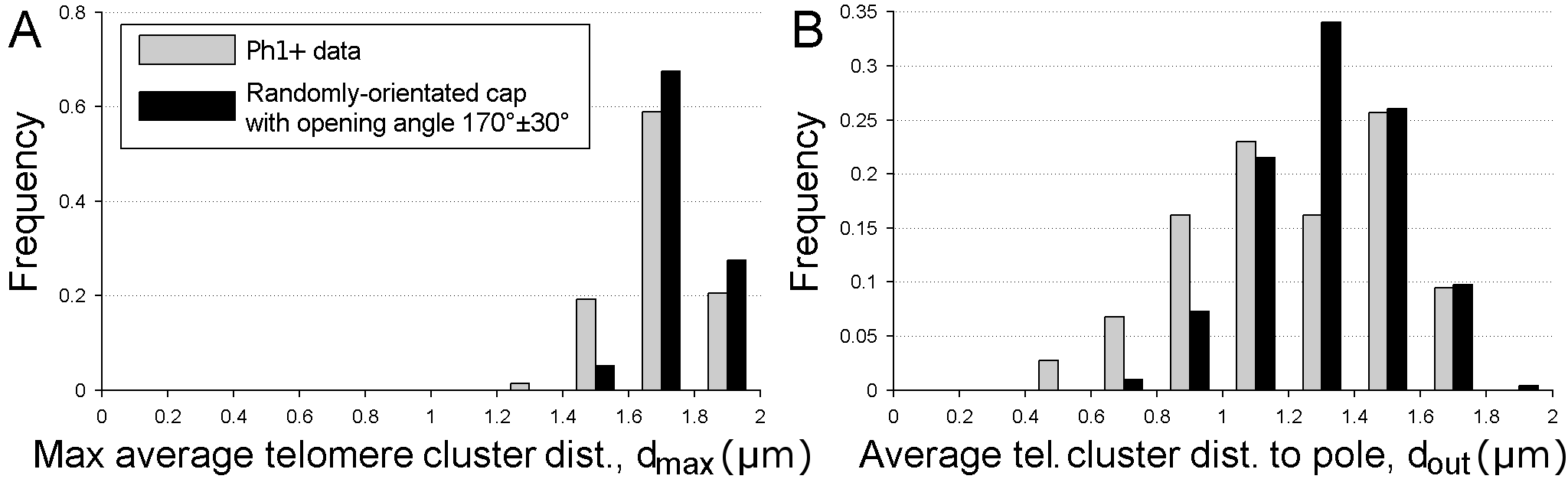

Supplement: Figure S2 — Histograms quantifying the telomere cluster spatial distribution in our Ph1+ meiocyte dataset (n = 74), compared to the theoretical situation where telomere clusters lie randomly positioned in a randomly-orientated cap subtending an opening angle of 170°±30°. A. Distribution of maximum average telomere cluster distance, d max (as a fraction of the nuclear radius). B. Distribution of average telomere cluster distance to outside pole, d out (as a fraction of the nuclear radius). (TIF) [file pcbi.1002812.s002.tif]

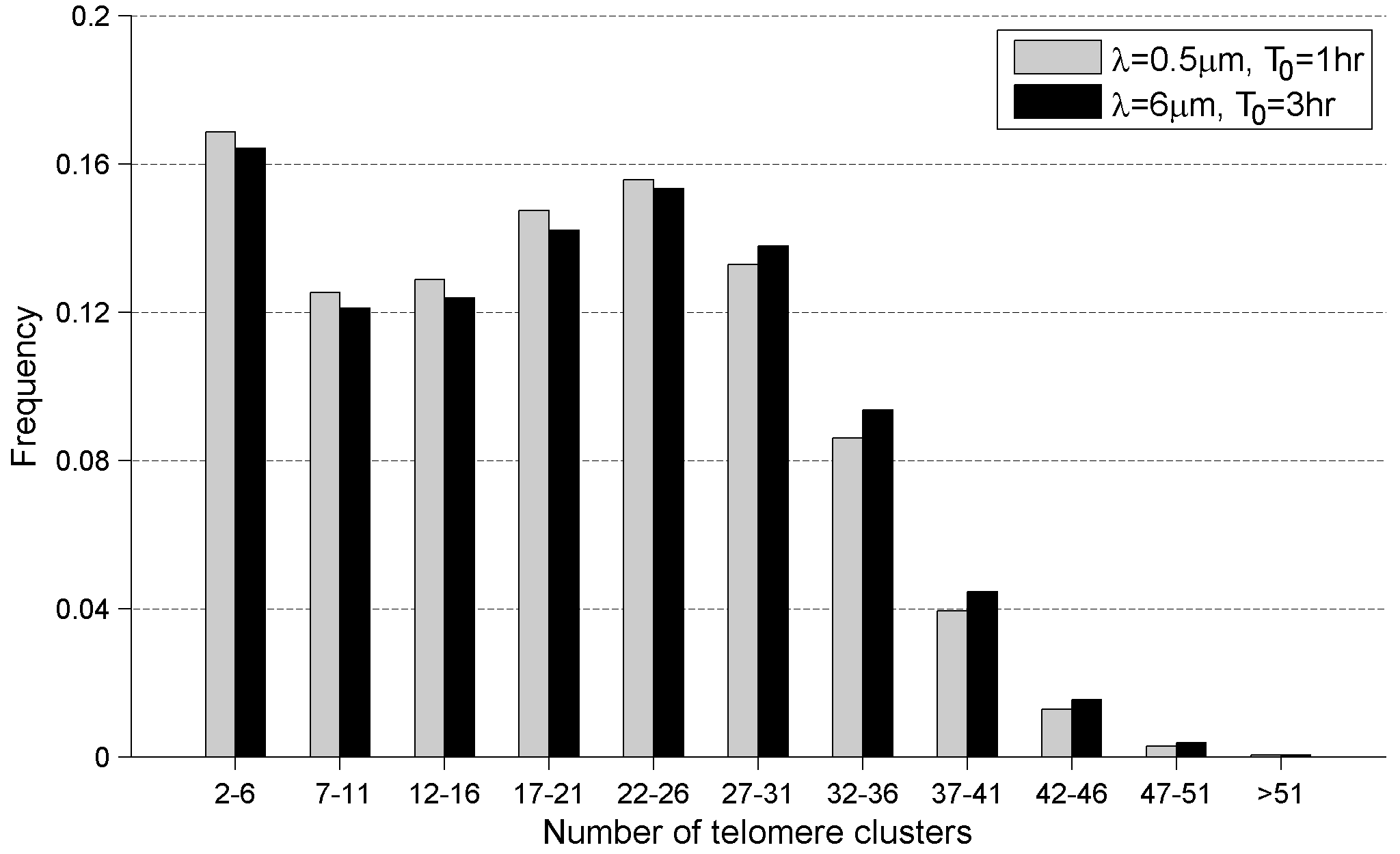

Supplement: Figure S3 — Comparison of the telomere cluster number distribution for two versions of the deterministic pure drift model with a randomly-orientated initial cap: one with λ = 0.5 µm, T 0 = 1 hr and one with λ = 6 µm, T 0 = 3 hr. (TIF) [file pcbi.1002812.s003.tif]

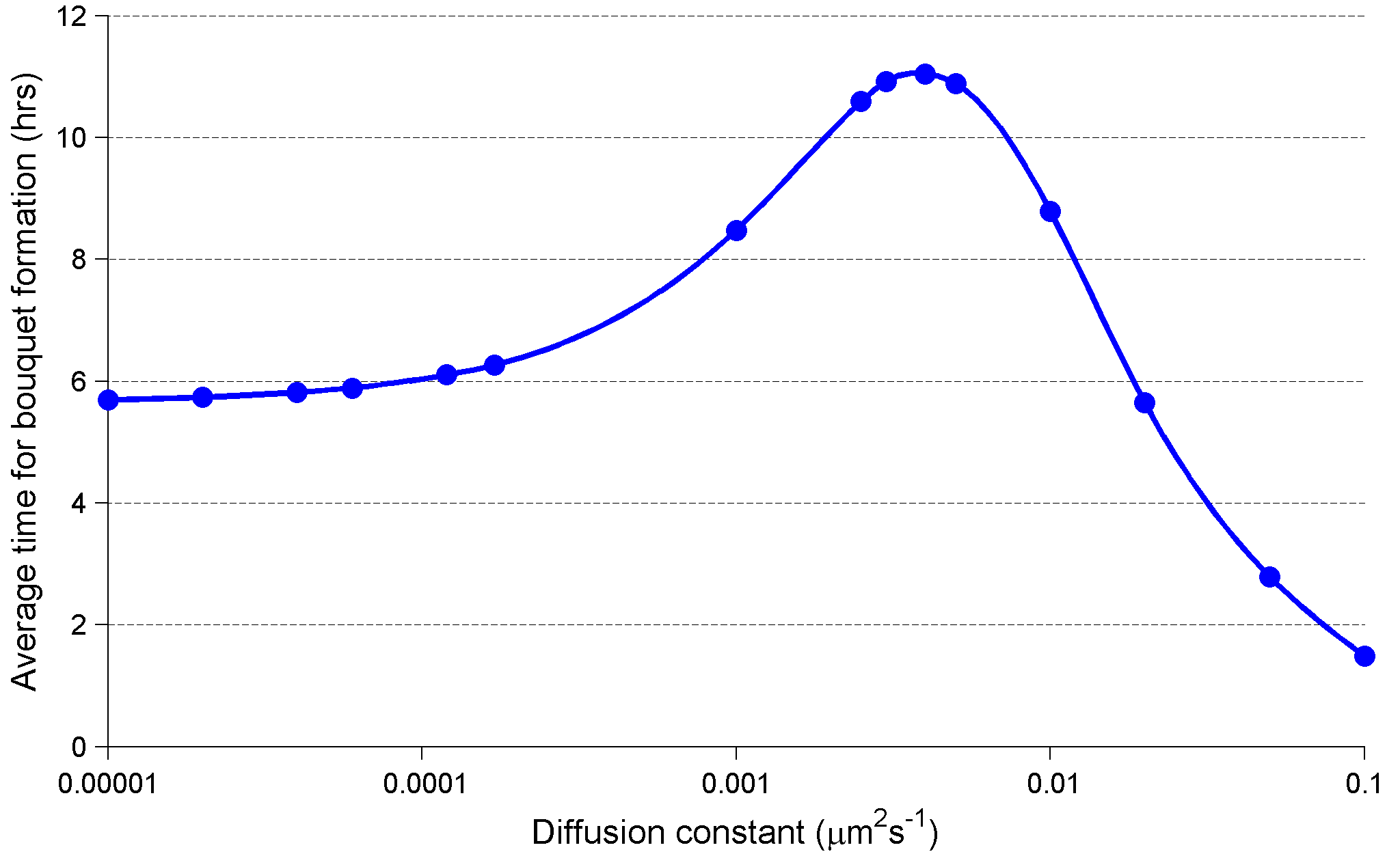

Supplement: Figure S4 — Average total time for bouquet formation against the diffusion constant for a constant drift speed of 8.5×10^−4^ µms−1. The error bars are too small to be visible. (TIF) [file pcbi.1002812.s004.tif]

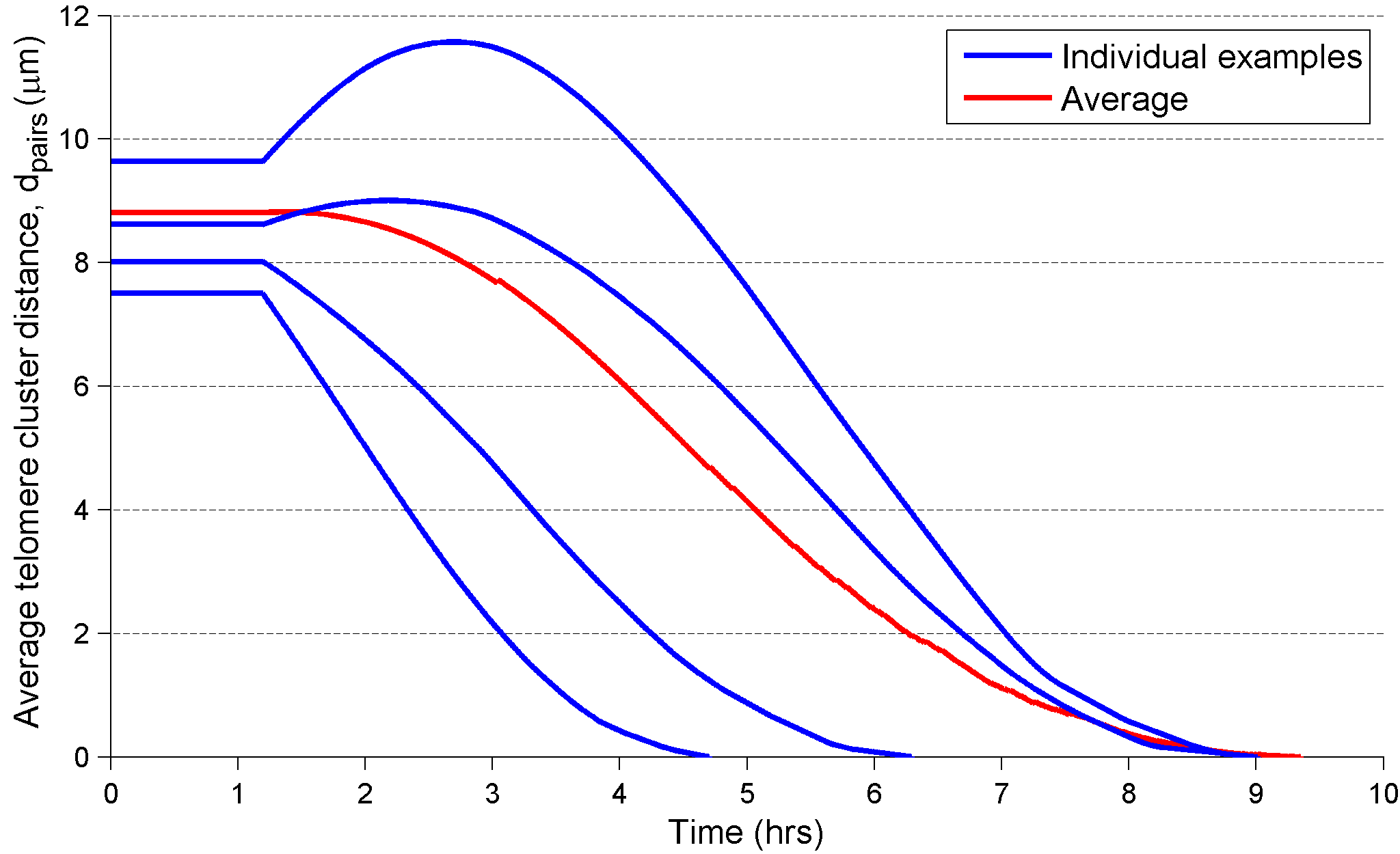

Supplement: Figure S5 — Behaviour in time of the average telomere cluster distance, d pairs, in the deterministic pure drift model with a randomly-orientated initial cap. Blue lines: individual examples. Red line: average over many initial conditions. (TIF) [file pcbi.1002812.s005.tif]
