# Supplementary material for: Quantitative Dynamics of Telomere Bouquet Formation
Source: PLoS Comput Biol. 2012 Dec 6;8(12):e1002812. doi: 10.1371/journal.pcbi.1002812 (PMC3516562; doi:10.1371/journal.pcbi.1002812)
Supplement: Text S1 — Important additional information related to the image analysis, the data analysis and the computational modelling. (DOC) [file pcbi.1002812.s006.doc]

**SUPPORTING INFORMATION FOR “Quantitative Dynamics of Telomere Bouquet Formation”**

**Details of image analysis**

The data used in the image analysis for each nucleus consisted of two 3D images (i.e. two z-stacks of 2D images): one showing the DNA (stained with DAPI) and one showing the telomeres (imaged with FISH).

First the boundary of the DNA was identified from the DNA image. For accuracy this was not fully automated. Rather, ellipses were drawn by eye around the DNA for three stacks: the lowest stack that contained a DAPI signal, the middle stack, and the highest stack that contained a DAPI signal. Then an ImageJ macro was used to fill in the remaining stacks by interpolating with an ellipsoid. This allowed a bitmask to be generated to describe the nucleus (the nucleus was defined as all points within the DNA boundary). Of course, the nucleus is likely to extend slightly beyond the edge of the DNA and this was taken into account by increasing the size of the nucleus by a fixed amount in all directions. We used 0.7µm for this fixed amount, although we checked that any value between 0 and 2µm made little difference to our results.

Next, a MATLAB script was written to identify the telomere cluster positions. The telomere image was first converted to a mask using a threshold value: only pixels above the threshold were considered part of a real telomere. For any given nucleus, the threshold value was chosen as 0.12 of the maximum pixel intensity. This value was chosen because it led to consistently-sized telomere clusters: values higher than this often missed telomeres that were clearly visible to the eye, and values lower than this would sometimes incorrectly identify large regions as telomere clusters. Then individual telomere clusters were identified and counted, with two pixels considered part of the same cluster if they were adjacent neighbours (diagonal neighbours were included such that, in three dimensions, each pixel had 26 adjacent neighbours). Once identified, the volume, the total intensity and the coordinates of the centre were recorded for each cluster. Clusters were only counted if their total intensity was greater than 2 (where each pixel had a maximum intensity of 1); this removed spurious background signals that were too small to represent real telomeres. Again we tried various values for the total intensity threshold (other than 2), but our results were largely unchanged. Finally, for each nucleus, the point on the nuclear membrane furthest from the anther centre was determined.

We attempted to check that we did not miss or artificially identify telomeres as follows. First, by comparing the telomere signal within nuclei to that well outside any nuclei (where there cannot be telomeres) we demonstrated that we are not incorrectly identifying the background signal as telomeres. Secondly, we calculated the total telomere signal within nuclei (i.e. the intensity sum over all clusters). Although this is only a very rough measure, we checked that the total intensity was indeed approximately constant (which is expected since the total number of telomeres within a nucleus is fixed). Thirdly, the fact that we never identified more than 56 telomere signals suggests that we are not incorrectly identifying non-telomeres.

**Definitions of the** **average telomere cluster distance to the outside pole, *d*_out_, and the maximum average telomere cluster distance, *d*_max_**

We define two measures of the spatial distribution of telomere clusters: *d*_out_ and *d*_max_. The first is the average intensity-weighted distance from the outside pole to the telomere clusters. Our image analysis identifies telomere clusters and calculates their size (how many pixels they occupy) and their intensity (the sum of the individual intensities of the pixels comprising the cluster). We define the outside of the nucleus as the point, $\vec{x}_{out}$, furthest from the centre of the anther. We then define *d*_out_ as

where *I*_i_ and $\vec{x}_{i}$ are the intensity and 3D vector position of cluster *i* respectively. Each sum runs over all telomere clusters. The second measure *d*_max_ is defined as the maximum average intensity-weighted distance from some point, $\vec{x}_{0}$, on the edge of the nucleus to all the telomere clusters, such that

The value of *d*_out_ ranges from 0 (if all the telomeres are at the outside pole) to 2*R* (if all telomeres are at the opposite “inside” pole), where *R* is the radius of the nucleus (assumed to be spherical). As bouquet formation progresses the value of *d*_out_ usually decreases since the telomeres normally move towards a point near the outside pole, where they join the bouquet. Conversely, *d*_max_ increases as the bouquet forms, reaching a maximum value when all telomeres are at one point. This maximum value would be 2*R* if telomeres are always confined to the nuclear envelope. However, since we do not know the exact position of the nuclear envelope, it is possible in some cases for *d*_max_ to take values larger than 2*R*. The minimum value of *d*_max_ is also unclear and depends again on exactly how the telomeres are confined. If telomeres are always exactly confined to lie on the nuclear envelope then the theoretical minimum value of *d*_max_ is 4*R*/3, which is attained in the limit of a very large numbers of telomeres distributed uniformly on the edge of the nucleus. However, if telomeres are allowed to leave the membrane and move into the nuclear interior, then the minimum value of *d*_max_ reduces to 6*R*/5. These minimum values both rise as the number of telomere clusters decreases. Since in our case we have around 28 telomere clusters with most (although not all) attached to the nuclear envelope, we can be sure that *d*_max_ cannot drop below 6*R*/5.

**Theoretical distributions of *d*_out_ and *d*_max_**

It is not immediately clear how to interpret the distributions of *d*_out_ and *d*_max_. For example, how would the distributions appear if the telomere clusters were confined to a randomly-orientated hemisphere or to an even smaller cap? To understand this we simulated various telomere cluster distributions and calculated the resultant *d*_out_ and *d*_max_ distributions. To do so, we chose some opening angle for the cap, randomly placed *n_0_* telomere clusters (chosen from a normal distribution with mean 27 and standard deviation 8 for tapetal cells and mean 26 and standard deviation 9 for *Ph1*+ meiocytes) on the surface of this cap, and calculated *d*_out_ and *d*_max_. By repeating this procedure for many (1,000) simulated nuclei we determined the theoretical distributions for *d*_out_ and *d*_max_. Since our data show that telomeres are not always exactly on the surface of the nucleus, we also considered the distributions of *d*_out_ and *d*_max_ that arise if we place telomeres near, but not always on, the surface of the cap. However, these distributions were not notably different to those obtained when all telomeres were placed exactly on the cap surface. Further, we also considered the case where the intensity of each cluster can vary, which accounts for the fact that clusters can contain different numbers of telomeres, but again this had negligible effect on the distributions.

When telomere clusters are distributed randomly throughout the surface of the entire sphere (with radius *R*), we find approximately normal distributions for both *d*_max_ (mean=1.51*R*, s.d.=0.07*R*) and *d*_out_ (mean=1.31*R*, s.d.=0.13*R*). However, if telomeres are confined within a hemisphere then *d*_max_ follows a normal distribution with a larger average (mean=1.72*R*, s.d.=0.05*R*). The behaviour of *d*_out_ in this case depends on whether the hemisphere is randomly orientated or not. For a hemisphere which always faces the outside of the sphere, *d*_out_ follows a narrow normal distribution with reduced average (mean=0.93*R*, s.d.=0.09*R*), whereas for a randomly-orientated hemisphere, the *d*_out_ distribution is still approximately normal, but is wider and has a larger average (mean=1.31*R*, s.d.=0.21*R*). A randomly-orientated hemisphere fits much better with the experimentally measured tapetal data (combining both *Ph1*+ and *Ph1*- cells), for which the *d*_max_ histogram has mean 1.74*R* and standard deviation 0.13*R* and the *d*_out_ histogram has mean 1.30*R* and standard deviation 0.33*R*. Similarly, a randomly-orientated hemisphere also fits well with the *Ph1*+ meiocyte dataset.

In fact, our data suggests that telomeres, for both tapetal cells and *Ph1*+ meiocytes, are contained in a slightly smaller region than a hemisphere, i.e. a cap subtending an opening angle slightly less than 180°. Further, if this opening angle is fixed then the distribution would be too narrow to fit with the data. We find the best fit corresponds to a randomly-orientated cap whose opening angle follows a normal distribution with mean 170° and standard deviation 30°.

**Details of synchrony within individual florets**

To determine whether individual florets showed synchrony in the time when bouquet formation began, we split our data from *Ph1*- meiocytes into the 12 individual florets that we measured and plotted the cluster number histogram for each. We then studied the width of these distributions, since individual florets should show a tighter distribution if bouquet initiation is synchronous. It is important to choose an appropriate measure of the width of each distribution. The standard deviation is not a fair test since, being a biased estimator, its value depends on the sample size. However, this is not the case for the variance, which is unbiased, and so can reliably be used to compare distributions of different sample sizes.

The variance of the whole data set is 160, whereas the mean variance for the 12 individual florets is only 66±18 (here the error is the standard error in the mean of the variance). To test whether this difference is significant we simulated sampling florets from the whole data set by randomly partitioning the full data set (159 nuclei) into 12 sets, with each set containing the number of nuclei of one of the real florets (i.e. {5, 12, 6, 22, 15, 16, 3, 6, 26, 10, 9, 29} nuclei). We then calculated the mean variance of these 12 distributions. By averaging over many (500,000) such partitions we found that the distribution of the mean variance due to random sampling had mean 160 and standard deviation 18 (i.e. 18 is the standard deviation of the mean of the variance). Since our measured mean variance of 66±18 is so far from the 160±18 expected if there is no synchrony, our result of synchrony within florets is highly significant.

The distribution of floret variances, with mean 66, also has a relatively large standard deviation of 61, suggesting that not all florets show complete synchrony. One possible explanation for this is that it is not florets that are synchronised, but the individual anthers within florets.

**Details of modelling**

To model bouquet formation the nuclear membrane was implemented as a 2D spherical surface of radius *R*, with each of the *n_0_* telomere clusters represented (in spherical polar coordinates) by a point (θ, φ) on the surface (0≤θ≤π, 0≤φ<2π). We chose *n_0_* from a truncated normal distribution with mean 32 and standard deviation 6, and rounded *n_0_* to the nearest integer. The truncation ensured that 2≤*n_0_*≤56.

The initial positions of the clusters were usually chosen to lie at random positions on a randomly-orientated cap. This was achieved by first choosing the opening angle of the cap from a normal distribution with mean 170° and standard deviation 30°. Then *n_0_* randomly positioned points were chosen to lie within this cap (centred at θ=0). Finally a random direction for the cap was chosen and all *n_0_* telomere clusters were rotated towards this random direction. Alternatively we also considered models where the initial telomere positions were taken from the real initial positions that we measured in *Ph1*+ meiocytes and tapetal cells (from both *Ph1*+ and *Ph1*- plants), although this made little difference to our analysis.

Before bouquet formation is initiated, telomere clusters remain in this initial configuration for some waiting time, *T*_0_. Of course, the telomeres are probably not fixed during this period and so possibly diffuse around in limited regions. However, despite this, the initial distribution of all telomeres is approximately fixed. To implement this feature, our simulations contain no dynamics until the time is greater than *T*_0_.

After a time *T*_0_, at each time step (Δ*t*=25s), each cluster both moved directly towards the bouquet site (drift) and diffused. For simplicity, we arranged that the bouquet site was always at the north pole (θ=0). This meant that drift was implemented simply by decreasing θ by *v*Δ*t*/*R* each time step, where *v* was the drift speed, whilst ensuring that θ never became negative. We deal with the fact that the bouquet does not always form exactly at the outside pole by rotating all telomeres by some angle before any measurements are taken. This angle is taken to fit the experimentally measured bouquet positions, i.e. the histogram in Figure S1. Since the clusters are confined to the nuclear membrane, diffusion is two-dimensional. Because of the difficulty with dealing with the (θ, φ) coordinate-system near the poles, diffusion for a given cluster was achieved by first rotating the cluster until it sat on the equator, then diffusing within a small, approximately flat patch on the equator, and finally rotating the cluster back. Within the equatorial patch, diffusion was implemented independently in both orthogonal directions as follows: a cluster diffused forwards a distance Δ*x* (=0.1µm) with probability *p*, backwards a distance Δ*x* with probability *p* and remained stationary with probability 1-2*p*. This probability was related to the diffusion constant via *p*=*D*Δ*t*/(Δ*x*)^2^. If a cluster moved within the bouquet radius, λ, of the pole (as measured along a great circle) then it was considered to have joined the bouquet, its θ was set to zero, and it was no longer permitted to move.

We collected data (number of clusters *N*, maximum average distance to a point on the membrane *d*_max_, average distance to outside pole *d*_out_) at randomly chosen times during each simulation. These times were chosen such that, during each second of the simulation, there was a 10^-5^ probability of collecting data, which corresponds, on average, to each simulation being sampled less than once. This corresponds well with our experimental data, where each cell was only imaged once.

As we only have ~100 images for each cell type that we study (*Ph1*+ meiocytes, *Ph1*- meiocytes, *Ph1*+/- tapetal cells) we also checked that 100 values of *N* are sufficient to fully sample the shape of the cluster number histogram (data not shown). Assuming that the underlying model is correct, we find that 100 images are indeed sufficient to fully capture the telomere dynamics.

**Insensitivity to changing the bouquet radius, λ**

Table 1 lists the parameters used in the model. The nuclear radius, *R*, is directly measured and the drift speed, *v*, is fixed by specifying the total time for bouquet formation. The mean and variation in the initial number of telomere clusters, <*n_0_*> and δ*n_0_*, and the waiting time, *T*_0_, are fit to the cluster number histogram (Figure 5). Also, the mean and variation of the opening angle for the initial telomere cap, <Θ_0_> and δΘ_0_, were fit to the *d*_max_ and *d*_out_ histograms.

This leaves the bouquet radius, λ, which specifies the radius of the region near the bouquet site within which a telomere cluster is considered to be part of the bouquet. We have no way of directly measuring λ and so it is important to understand its effect on telomere dynamics. Whenever λ is changed it is important to refit *T*_0_ to ensure that the total time for bouquet formation is unchanged. When this is done we find almost no change to our results for a wide range of values for λ. In particular, varying λ between 0.5μm and 6μm makes practically no difference to either the total time for bouquet formation, the standard deviation of this time, or the shape of the cluster number histogram. For example, see Figure S3, which compares the cluster number histogram for λ=0.5μm and λ=6μm. Although this is a relatively large change in λ, it is not surprising that there is little change to our results: the shape of the cluster number histogram is largely governed by the initial distribution of telomeres and not the details of when they reach the bouquet.

**Bouquet formation time is a complicated function of the diffusion constant**

As we show, a pure drift model fits our observed telomere data well, without the need for diffusion. However, it is nevertheless interesting to examine the effect that adding diffusion has on the total time for bouquet formation, *T*_bouq_. To this end, we fixed the drift at 8.5×10^-4^μms^-1^ and varied the diffusion constant, *D*, from 0 to 0.1μm^2^s^-1^ (Figure S4).

As the diffusion constant is increased from zero, the total time for bouquet formation initially increases due to the difficulty the final few telomeres have in joining the bouquet (with diffusion, unlike for pure deterministic drift, it is possible for a telomere to “miss” the bouquet). However, at a certain value of *D*, which is about 0.004μm^2^s^-1^ with the above value of drift, *T*_bouq_ reaches a maximum. For values of *D* above this, the difficulty due to telomeres “missing” the bouquet is outweighed by the decreased time of searching for the bouquet due to fast diffusion. As *D* is increased further, *T*_bouq_ continues to decrease, such that *T*_bouq_→0 as *D*→∞.

Thus, for many values of *T*_bouq_ there are in fact two models which match the observed total time, with different values of the diffusion constant. Although it is possible that the second model with the higher diffusion constant could be generated by a disordered cytoskeleton, such a model would again lead to large variations (over 40%) in the total time for bouquet formation, which are not observed experimentally.

**Extensions to the model**

To check whether other effects could qualitatively change our results we considered various extensions to our basic model, although in all cases we found little improved matching with the experimental data.

First, we implemented a hard radius for telomere clusters, such that two telomere clusters could not approach closer than twice the hard radius. Separately we implemented a binding radius, such that if two telomere clusters moved within this radius then they formed a bound cluster which could never disassociate. This would be relevant if telomeres have an affinity for each other. Our analysis indeed suggests that this may be the case since, even in tapetal cells, the initial cluster count is only about half the number of sister chromatid telomeres. However, neither a binding radius nor a hard radius made a significant difference to our analysis.

Secondly, we implemented a resolution distance to account for our inability to resolve sufficiently nearby telomere clusters using our microscopy setup. If two or more telomere clusters moved within this resolution distance then they appeared as one cluster. At later times it was possible for such a group of telomere clusters to disperse (e.g. via diffusion) and appear as multiple signals again. Again this effect made almost no difference to the simulated dynamics of bouquet formation.

We also considered a model where the diffusion constant depended on the cluster size, such that the diffusion constant for a cluster containing *m* telomeres was *D*/*m* for constant *D*. This captures the fact that clusters with more telomeres are connected to a greater mass of DNA and may therefore diffuse more slowly. For a similar reason, we simultaneously reduced the drift speed to *v*/*m* for constant *v*. Although these effects may be present, they do not lead to a better fit to the data, and are in any case irrelevant for the pure drift model where telomeres only cluster when they reach the bouquet site.

We also considered models studying what would happen if our analysis sometimes missed telomere clusters. Each time a snapshot of the system was obtained from our simulations, we implemented a fixed probability that each telomere cluster would not be observed. Again, for realistic probabilities (up to 0.1), this made little difference to the bouquet dynamics.

The simplest version of the model involves a constant drift speed, a constant radius, and a constant waiting time. However, it is more realistic to allow fluctuations in these parameters. A fluctuating drift speed was incorporated by choosing, at each time step, the drift speed, *v*, from a normal distribution with mean 8.5×10^-4^μms^-1^, truncated to ensure that *v* is always positive. Various standard deviations were considered (up to about 4×10^-4^μms^-1^), although there was little improved matching with the experimental data. The fluctuations in the radius were introduced by choosing an independent radius (from a normal distribution with mean 8μm and standard deviation 4μm) for each simulation. However, after averaging over many nuclei, there was again no improvement in the experimental fit. Fluctuations in the waiting time (with mean of 72 minutes and standard deviation up to 72 minutes) were implemented in a similar manner and found, as expected, to have no effect on the cluster number histogram.

Finally, our simple model assumed that the cytoskeleton is arranged so that telomeres move directly towards the bouquet site. It is possible that the arrangement is more complicated than this. For example, we also considered a model where telomeres first move along lines of constant latitude until encountering either φ=0 or φ=π, and then move directly towards the bouquet site. Perhaps surprisingly, this alteration also made almost no difference to the shape of the cluster number histogram.

**Goodness of fit testing of cluster number histogram with model**

Although the pure drift model appears to match well with the *Ph1-* data (Figure 5), it is important to test this rigorously. To do so we performed a chi-squared goodness of fit test, which, since the cluster number is discrete, is more appropriate than a Kolmogorov-Smirnov test. This allowed us to test the hypothesis that the data is well-described by the model (the null hypothesis) against the converse (the alternative hypothesis).

Since the chi-squared test does not perform well with bins containing fewer than 5 data points, we excluded the final few bins and only tested the distribution up to *N*=41. This gave a chi-squared test statistic of 2.1. The numbers of degrees of freedom is given by the number of bins (8) minus the number of fitting parameters. Our model contains four parameters that must be fit before the data can be compared: the total area under the histogram, the waiting time *T*_0_, the mean initial number of telomere clusters <*n_0_*>, and the standard deviation of the initial number of telomere clusters, δ*n_0_*. So we are left with four degrees of freedom. At the 5% confidence limit and 4 degrees of freedom the relevant value to compare the test statistic to is 9.5. Since this is larger than our calculated 2.1, there is no reason to reject the null hypothesis that the data is well-described by the pure deterministic drift model.

**Adding a probabilistic drift direction to the model**

In the simplest version of the model telomeres move directly towards the position of the bouquet site, presumably due to movement along some cytoskeletal element. However, although the relevant cytoskeletal elements may preferentially point towards the future bouquet site, there may be variation in the drift direction, as discussed in the main text.

To model this we implement a spread in telomere drift directions. Rather than model in detail the structure and network of the cytoskeleton, we simply assume that the effect of the cytoskeleton is to enforce a local drift direction on each telomere. Telomeres move in this local direction for some fixed distance, called the run length, *L*_R_, which we normally take as 1μm, before a new direction is chosen, with the process repeating.

So, for each telomere cluster, we randomly and independently pick a drift direction, i.e. each cluster is now characterised not only by its position (θ, φ), but also by its bearing ψ. For simplicity, and in the absence of any better model, the drift direction is chosen from a Gaussian distribution which is centred on 0° (direct movement to the bouquet site) and with a fixed standard deviation, δψ. The distribution is truncated to ensure a bearing between -180° and 180°. At each time step, a telomere cluster’s position is updated by moving a distance *v*Δ*t* along a great circle with initial bearing ψ. In addition the cluster’s bearing is updated to point along the same great circle at the end of the time step as at the beginning. This is important since the bearing continually changes along a great circle. After a number of time steps, when the cluster has moved along one great circle for a distance of *L*_R_, a new random bearing is chosen and, in subsequent time steps, the cluster will move along a new great circle. As before, a telomere cluster ceases to move and is considered part of the bouquet when it moves within the bouquet radius of the bouquet site.

As with the case without a random drift direction, for each pair of values of δψ and *L*_R_ we fit both the drift speed *v* (to ensure the bouquet forms in the correct total time) and the waiting time *T*_0_ (to ensure the local maximum around *N*=28 has the correct height relative to the peak at *N*=2–6). To compare the cluster number histograms between different values of δψ, we compute the chi-squared test-statistic (as explained above). This allows us to examine the value of δψ. With δψ=0° (the simplest version of the model with drift directly towards the bouquet site), this statistic is 2.1. However, for *L*_R_=1μm, as δψ is increased the statistic decreases, reaching a minimum of 1.6 when δψ=40°, before increasing as δψ is further increased. If *L*_R_=0.5μm is used the minimum statistic (now with value 1.9) is obtained at a similar value of δψ. These statistics are better, though only marginally, than a directed movement model with δψ=0°. Hence, even without knowing the exact run length, we can predict that significant cytoskeletal disorder does not disrupt the bouquet formation dynamics.
